# Supplementary material for: Improvement in quality of life and cognitive function in Post-COVID syndrome after online occupational therapy: Results from a randomized controlled pilot study
Source: PLoS One. 2025 May 20;20(5):e0312714. doi: 10.1371/journal.pone.0312714 (PMC12091760; doi:10.1371/journal.pone.0312714)
Supplement: S1 Table — (DOCX) [file pone.0312714.s001.docx]

***S1 Table. Differences between study arms adjusted for baseline scores (cases with missing follow-up data excluded)***

|  | **T2 difference**  **(95% CI)^1^** | **T3 difference**  **(95% CI)^1^** | **P T2^2^** | **P T3^2^** |
| --- | --- | --- | --- | --- |
| **d2-R^3^** | | | | |
| **video vs. control** | 0.02 (-0.58 – 0.62) | 0.06 (-0.67 – 0.78) | 1.00 | 0.921 |
| **interactive vs. control** | 0.16 (-0.34 – 0.67) | 0.31 (-0.30 – 0.93) | 1.00 | 0.627 |
| **WIT-2^3^** | | | | |
| **video vs. control** | 0.29 (-0.30 – 0.88) | -0.38 (-1.11 – 0.36) | 0.462 | 0.649 |
| **interactive vs. control** | 0.30 (-0.17 – 0.77) | -0.08 (-0.58 – 1.16) | 0.372 | 0.833 |
| **EQ-5D-3L index^3^** | | | | |
| **video vs. control** | 0.03 (-0.16 – 0.22) | -0.06 (-0.29 – 0.16) | 0.700 | 0.797 |
| **interactive vs. control** | 0.16 (0.01 – 0.32) | 0.06 ( -0.11 – 0.23) | 0.031 | 0.797 |
| **EQ VAS^3^** | | | | |
| **video vs. control** | 3.28 (-11.85 – 18.4) | 2.29 (-22.0 – 26.5) | 0.838 | 1.00 |
| **interactive vs. control** | 10.06 (-6.17 – 26.3) | 9.66 (-11.9 – 31.3) | 0.389 | 0.806 |
| **Neuro-QoL™ v2. 0 cognitive function short form^3^** | | | | |
| **video vs. control** | -0.32 (-4.20 – 3.55) | -0.18 (-6.90 – 6.54) | 0.84 | 1.00 |
| **interactive vs. control** | 4.70 (0.90 – 8.51) | 2.41 (-3.81 – 8.64) | 0.011 | 1.00 |
| **IMET^4^** | | | | |
| **video vs. control** | 12.22 (-4.25 – 28.69) | 4.12 (-23.50 – 31.80) | 0.139 | 0.920 |
| **interactive vs. control** | -9.32 (-27.16 – 8.53) | -9.41 (-32.40 – 13.60) | 0.196 | 0.920 |
| **COPM performance** | | | | |
| **video vs. control** | 0.53 (-0.37 – 1.43) | - | 0.462 | - |
| **interactive vs. control** | 0.35 (-0.44 – 1.14) | - | 0.554 | - |
| **COPM satisfaction** | | | | |
| **video vs. control** | 0.39 (-0.75 – 1.53) | - | 1.00 | - |
| **interactive vs. control** | 0.22 (-0.88 – 1.31) | - | 1.00 | - |

^1^differences and CI adjusted according to baseline values employing repeated measurement variance analyses, ^2^Bonferroni Holm adjusted for multiple testing, ^3^positive value: improvement, ^4^ negative value: improvement, -: not assessed at T3.
