# Supplementary material for: Improvement in quality of life and cognitive function in Post-COVID syndrome after online occupational therapy: Results from a randomized controlled pilot study
Source: PLoS One. 2025 May 20;20(5):e0312714. doi: 10.1371/journal.pone.0312714 (PMC12091760; doi:10.1371/journal.pone.0312714)
Supplement: S1 File — (PDF) [file pone.0312714.s002.pdf]

German version of study protocol and supplementary material and amendments

“Improvement in quality of life and cognitive function in Post Covid Syndrome after online occupational therapy: results from a 3 randomized controlled pilot study “ by Schröder et al.

This document contains

- Study protocol (pages 2-15)
- Supplementary material (pages 16-38)
- Amendments to the study protocol (pages 39-41)

Ethik- Kommission der Medizinischen Hochschule Hannover
(nachrichtlich an die Ethik- Kommission der Universitätsmedizin Göttingen)

<u>Studienprotokoll</u>

für die Studie

ErgoLoCo – Digitale ergotherapeutische Interventionsentwicklung für Long COVID-PatientInnen

Klinik für Rheumatologie und Immunologie
Medizinische Hochschule Hannover

gemeinsam mit

Institut für Allgemeinmedizin Göttingen

und

Ostfalia Hochschule für angewandte
Wissenschaften Braunschweig

Studienleitung

PD Dr. med. Alexandra Jablonka
Klinik für Immunologie und Rheumatologie - OE 6830
Medizinische Hochschule Hannover
Carl Neuberg Straße 1
30625 Hannover
Email: Jablonka.Alexandra@mh-hannover.de
Tel: +49 511 532 3014

AntragstellerInnen

UMG:	MHH:	Ostfalia:
Dr. med. Frank Müller	PD Dr. med. Alexandra Dopfer-Jablonka	Prof. Dr. Frank Klawonn
Prof. Dr. Eva Hummers	Prof. Dr. med. Georg Behrens	
	Prof. Dr. med. Sandra Steffens	
	Prof. Dr. med. Christine Happle, PhD	

Hannover, Göttingen, Wolfenbüttel, Juni 2022

Inhalt

1. Zielsetzung und Begründung der Studie ... 4

1.1 Stand der Forschung ... 4

1.2 Begründung für die durchzuführende Studie: Nutzen für die Heilkunde bzw. wissenschaftlicher Erkenntniswert ... 5

1.3 Ziele und wissenschaftliche Fragestellungen ... 6

2. Studienzweige und Studienüberblick ... 6

3. **Beschreibung der Intervention** ... 7

4. Nicht verblindete randomisierte kontrollierte Pilotstudie ... 8

4.1 Charakterisierung der TeilnehmerInnen ... 8

4.2 Rekrutierung ... 8

4.3 Aufklärung und Einwilligung ... 9

4.4 Teilnahme und Widerruf ... 9

4.5 Studienablauf – Vorgehen und Durchführung ... 9

4.6 Pseudonymisierung und Datenschutz ... 11

5. Liste der Anhänge ... 13

6. Literaturverzeichnis ... 14

7. Anhänge ... 16

Anhang 1: Aufklärung Jugendliche ab 16. Lebensjahr (inklusive Elternaufklärung) ... 16

Anhang 2: Aufklärung Erwachsene ... 22

Anhang 3: Einverständniserklärung Jugendliche ab 16. Lebensjahr (inkl. Elterneinverständniserklärung) ... 28

Anhang 4: Einverständniserklärung Erwachsene ... 31

Anhang 5: Angaben zum Testinstrument COPM ... 33

Anhang 6: Angaben zum Testinstrument WIT-22 ... 33

Anhang 7: Angaben zum Testinstrument IMET ... 34

Anhang 8: Angaben zum Testinstrument NeuroQual ... 35

Anhang 9: Fragen zur Prozessevaluation ... 36

1. Zielsetzung und Begründung der Studie

Long COVID ist ein während der aktuellen Pandemie neu entstandener Sammelbegriff für Langzeitsymptome, die nach COVID-19 persistieren oder auftreten. Die Pathomechanismen, die zu Long COVID führen, sind bislang noch nicht klar, wodurch Möglichkeiten des therapeutischen Vorgehens sehr limitiert sind. Long COVID zeigt sich in Symptomclustern, die sich meist überlappen und in ihrer Intensität schwanken (Wong-Chew et al. 2022). Das produktive und soziale Leben Betroffener, sowie deren subjektives Wohlbefinden sind bei Long COVID häufig und über längere Zeiträume stark beeinträchtigt. Untersuchungen zu Prävalenz und klinischen und sozioökonomischen Aspekten von Long COVID zeigen, dass Long COVID PatientInnen starke Beeinträchtigungen des Sozial- und Familienlebens zeigen (Nittas et al. 2022).Eines der Hauptsymptome bei Menschen nach durchgemachter COVID-19 Erkrankung ist eine verminderte kognitive Leistungsfähigkeit, die sich hauptsächlich als „Brain Fog", also Konzentrationsschwierigkeiten und Fatigue zeigt. Diese Symptome können zu Problemen der Bewältigung alltäglicher Aktivitäten führen und die Wiederherstellung des Gesundheitszustands signifikant beeinträchtigen (Hugon et al. 2022)

Derzeit sind noch keine evidenzbasierten Behandlungsmöglichkeiten für Long COVID bekannt. Empfohlen wird, z.B. durch das britische National Institute for Health and Care Excellence (NICE), eine Integration multidisziplinärer Behandlungsmethoden, die auf Symptommanagement und Verminderung funktioneller Einschränkungen abzielen (NICE 2022).Ergotherapie gilt dabei als integraler Bestandteil eines solchen multimodalen, rehabilitativen Behandlungskonzepts [AOTA: Occupational therapy in the age of Coronavirus] (Margetis et al. 2021; AOTA 2021; Belli et al. 2020). In Deutschland kann Ergotherapie durch ÄrztInnen als Heilmittel für die Behandlung von Long COVID über einen Zeitraum von zwölf Wochen verordnet werden (KBV, 2021). Studien zeigen die Wirksamkeit von Ergotherapie in verschiedenen klinischen und rehabilitativen Settings zur Behandlung von Alltagseinschränkungen und Symptomen, die denen von Long COVID entsprechen (Kos et al. 2016).

Auf dieser Grundlage und durch partizipative Einbindung von Stakeholdern (Betroffene, deren Angehörige, medizinisches Fachpersonal) wurde für die aktuelle Studie eine digitale ergotherapeutische Intervention zur Behandlung von Alltagsbeeinträchtigungen bei Long COVID entwickelt, die im Rahmen des hier beantragten Studienvorhabens erprobt und evaluiert werden soll. Die neue Behandlungsform soll sich an Jugendliche sowie Erwachsene richten. Die Ergotherapie soll für eine Interventionsgruppe als live-online Ergotherapie und für eine weitere via zuvor aufgenommenen Videos vermittelt werden. Vor und nach der Intervention sollen bei den ProbandInnen Testungen der kognitiven Fähigkeiten und Befragungen zur Lebens- und Handlungsqualität stattfinden.

1.1 Stand der Forschung

1.1.1 *Long COVID bei Erwachsenen, Kindern und Jugendlichen*

Der Begriff Long COVID beschreibt Symptome, die mehr als vier Wochen nach Beginn einer SARS-CoV-2 Infektion fortbestehen oder neu auftreten (Koczulla et al. 2021). Als Post COVID werden gemeinhin Symptome benannt, die im Zusammenhang mit einer akuten SARS-CoV-2 Infektion oder danach auftraten, mehr als zwölf Wochen später noch vorliegen, mindestens zwei Monate andauern oder wiederkehrend und in wechselnder Stärke auftreten und nicht anderweitig erklärt werden können (Nalbandian et al. 2021) Eine systematische Übersichtsarbeit zeigte 55 verschiedene Langzeiteffekte bei COVID-19 auf (Lopez-Leon et al. 2021) Als häufige Symptome wurden hierbei Atemnot, Kopfschmerzen, Husten, Ermüdung und kognitive Beeinträchtigungen im Sinne von Brain-Fog

identifiziert. Anhaltende Symptome nach COVID-19 Erkrankung können erhebliche Auswirkungen auf die Rückkehr in Alltag und Beruf bedeuten, was häufig auch finanzielle Folgen nach sich zieht (Davis, et al., 2021). Eine aktuelle Studie aus den Niederlanden zeigt, dass auch mehr als ein Drittel aller Kinder und Jugendlichen mit Long- oder Post-COVID schwere Beeinträchtigungen im Alltag aufgrund von Konzentrationsschwäche zeigen (Brackel et al. 2021). Auch eine Studie aus Großbritannien bestätigt, dass diese Symptome häufig bei Jugendlichen auftreten (Miller et al. 2021). Bislang gibt es keine evidenzbasierte Therapie für diese Symptome, weder für Jugendliche noch für Erwachsene.

1.1.2 Ergotherapeutische Interventionen bei Long COVID

Aktuelle Leitlinien in Deutschland und international empfehlen ganzheitliche, personenzentrierte Ansätze zu Befunderhebung und Behandlung der von Long COVID betroffenen PatientInnen. Die NICE-Guidelines verweisen hierbei auf die Anwendung multidisziplinärer Rehabilitationsansätze zur Förderung von Symptommanagement und Minimierung funktioneller Einschränkungen. Insbesondere soll hierbei berücksichtigt werden, dass anhaltende Symptome Alltagsleben und tägliche Aktivitäten wie z.B. Arbeit, Bildung, Mobilität, Selbstständigkeit sowie das psychologische Wohlbefinden beeinträchtigen (NICE 2022). Hierbei kann der Ergotherapie eine besondere Rolle zukommen. Ergotherapie unterstützt Menschen jeden Alters dabei, wichtige Alltagsrollen und Aktivitäten, die aufgrund funktioneller Beeinträchtigungen eingeschränkt sind, wiederzuerlangen oder zu kompensieren (DVE). Dies beinhaltet unter anderem die Wiederherstellung der Handlungsfähigkeit sowie das Wiederaufgreifen existenzieller und verantwortungsvoller Aufgaben, beispielsweise Rückkehr an Arbeits- oder Ausbildungsplatz bzw. in die Schule. In der Ergotherapie wird ein besonderer Fokus auf Identifikation neuer Handlungs- und Teilhabestrategien gelegt, die es PatientInnen ermöglichen, alltägliche Aktivitäten in den verschiedenen Lebensbereichen wiederzuerlangen bzw. aufrecht zu erhalten (Le Granse et al.). Neue Daten zeigen, dass Ergotherapie bei Long COVID effektiv zur Überwindung von Handlungsproblemen im Alltag beiträgt (Vij 2021).

1.2 Begründung für die durchzuführende Studie: Nutzen für die Heilkunde bzw. wissenschaftlicher Erkenntniswert

Die Entwicklung geeigneter Therapiemethoden zur Behandlung von Long COVID ist von akuter und zentraler Wichtigkeit, da die Erkrankung bei einer zunehmenden Zahl an PatientInnen zu langfristigen Einschränkungen in allen Lebensbereichen mit besonderem Einfluss auf Lebensqualität und produktives Handeln führt (Townsend et al. 2020). Long COVID ist eine vergleichsweise neue Erkrankung, sodass die Prävalenz aktuell noch nicht genau eingeschätzt werden kann. Schätzungen gehen jedoch von einem Risiko von ca. 7% aller Infizierten aus (Al-Aly et al. 2022), sodass ÄrztInnen weltweit sich nun bereits mit einer hohen Zahl von PatientInnen konfrontiert sehen und auch in den kommenden Jahren mit einer hohen Anzahl von Betroffenen zu rechnen ist.

Die aktuell beantragte Studie soll eine digitale Intervention zur Behandlung dieser PatientInnen evaluieren. Das Fortschreiten der Digitalisierung bringt neue Herausforderungen und Chancen für den Gesundheitssektor mit sich, die auch die Entwicklung und das Angebot zeitgemäßer und ressourcensparender, kosteneffizienter Gesundheitsdienstleistungen beinhalten (Walzer 2022). Durch die aktuelle pandemische Lage wurden seit 2020 teletherapeutische Angebote im Gesundheitswesen vermehrt eingesetzt, wodurch breite PatientInnengruppen erreicht und erfolgreich behandelt werden konnten (Kataria und Ravindran 2018). Deutlich wurde dabei jedoch, dass eine gezielte und evidenzbasierte Entwicklung strukturierter telemedizinischer Methoden notwendig ist, um die Qualität digitaler Behandlungsangebote zu sichern (Peine et al. 2020).

Im Zusammenhang mit der Behandlung von Long COVID ist zu erwarten, dass digitale Behandlungsoptionen ein niederschwelliges und gut angenommenes Angebot darstellen können,

durch das Betroffene in ihrem direkten Alltagsumfeld versorgt werden können. Faktoren wie Mangel personeller Kapazitäten, regionale Unterversorgung sowie Fachkräftemangel in den Gesundheitsfachberufen können durch digitale Vermittlung zumindest teilweise entschärft werden.

Die Evaluation einer digitalen ergotherapeutischen Intervention bei Long COVID erscheint daher als sinnvoll und wichtig. Sollte die Intervention wirksam in der Behandlung von Long COVID sein, wäre dies für viele PatientInnen in Deutschland und darüber hinaus hilfreich und würde signifikant zur Reduktion von Morbidität und Versorgungskosten im Zusammenhang mit COVID-19 beitragen.

1.3 Ziele und wissenschaftliche Fragestellungen

Ziel dieser Studie ist die Evaluation einer digitalen, ergotherapeutischen Intervention zur Behandlung von Alltagsbeeinträchtigungen aufgrund von Long COVID-assoziierten kognitiven Störungen wie Brain Fog, Konzentrationsstörungen und Fatigue. Konkret sollen folgende Hypothesen überprüft werden,

1. Wird eine online vermittelte Ergotherapie-Intervention von Long COVID PatientInnen angenommen?
2. Führt eine online vermittelte ergotherapeutische Intervention zur Reduktion von objektiv messbaren und subjektiv wahrgenommenen Long COVID assoziierten Handlungseinschränkungen im Alltag?
3. Führt eine online vermittelte ergotherapeutische Intervention zur Reduktion von objektiv messbaren und subjektiv wahrgenommenen kognitiven Einschränkungen?
4. Welche Faktoren sind bei dieser digitalen ergotherapeutischen Intervention wirksam, und welche behindern deren Erfolg?

2. Studienzweige und Studienüberblick

Die Pilotstudie soll als eine randomisierte, kontrollierte, unverblindete Interventionsstudie durchgeführt werden.

Sie soll in zwei gleich großen Gruppen durchgeführt werden: n=80 Erwachsene im Alter zwischen 30 und 50 Lebensjahren (n= 20 sollen live online Ergotherapie erhalten, n=20 sollen Ergotherapie mittels zuvor aufgenommener, on-demand abrufbarer Videos erhalten, n=40 Kontrollen sollen keine Therapie erhalten) sowie n=80 Jugendliche im Alter zwischen 16 und 18 Lebensjahren (n= 20 sollen live online Ergotherapie erhalten, n=20 sollen Ergotherapie mittels zuvor aufgenommener, on-demand abrufbarer Videos erhalten, n=40 Kontrollen sollen keine Therapie erhalten).

Die Rekrutierung der ProbandInnen, sowie Inklusion, Testung und Therapie sollen online über geschützte, digitale Kanäle stattfinden. Alle n=160 ProbandInnen erhalten zu drei Zeitpunkten kongnitive Testungen: Startzeitpunkt bei Inklusion (t0), zwölf Wochen später (t1) und zu Woche 24 (t2, Studienende). Die Randomisierung in die verschiedenen Behandlungsgruppen findet zu t0 nach der ersten kognitiven Testung statt.

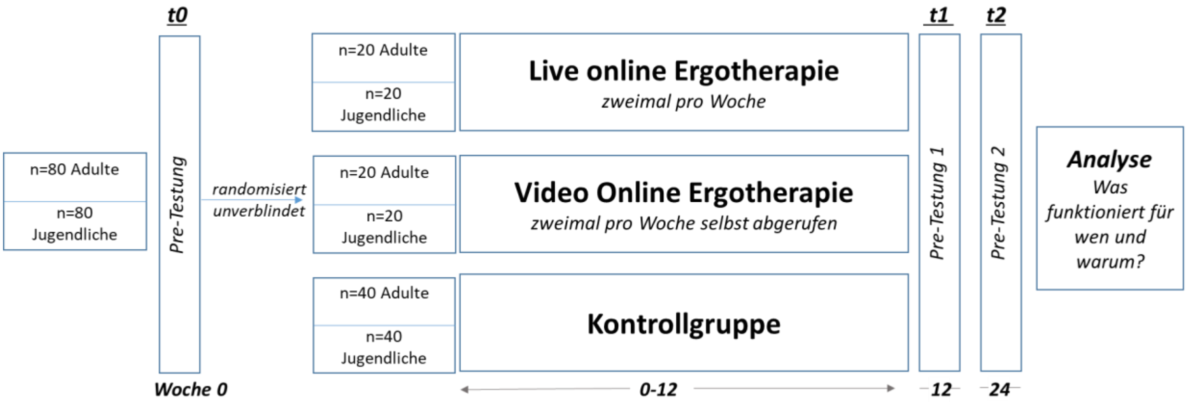

***Abb. 1:** Übersicht über die geplante Intervention. n=160 ProbandInnen sollen online rekrutiert werden. Zu Zeitpunkt (t) 0 erhalten sie eine kognitive Testung und Analyse ihrer Fähigkeiten und Beschwerden im Alltag und sollen dann in die Studienzweige randomisiert werden. Die Interventionsgruppen erhalten jeweils zweimal wöchentlich Ergotherapie (live online oder über zuvor aufgenommene Videos). Zwölf (t1) und 24 Wochen (t2) später werden alle ProbandInnen auf Ihre kognitiven Fähigkeiten und Beschwerden im Alltag erneut getestet und über ihre Erfahrungen mit der Intervention befragt.*

3. Beschreibung der Intervention

Die Interventionsentwicklung erfolgte auf Grundlage des Medical Research Council Framework (Skivington et al., 2021). Mit einer auf diesem Framework basierten, strukturierten Vorgehensweise wird sichergestellt, dass die Intervention Zielgruppengerecht gestaltet und dem aktuellen Evidenzstand basiert und Erfahrungen von ExpertInnen mit einbezieht.

Die Intervention folgt einer Modulstruktur von sechs aufeinanderfolgenden Therapiemodulen, die aus jeweils vier Interventionseinheiten von 30 Minuten bestehen. Jedes Modul besteht aus der Kombination von durch ErgotherapeutInnen angeleiteten Therapieeinheiten und Anleitungen zum Selbsttraining. Teilnehmende beider Interventionsgruppen schließen nach Beendigung des letzten Moduls einen vollständigen ergotherapeutischen Prozess inklusive Evaluation, Analyse, Adaptation und Re-Evaluation der Betätigungsproblematik ab.

Die Intervention soll über das Online-Portal der bereits etablierten digitalen Corona-Plattform DEFEAT der Medizinischen Hochschule Hannover vermittelt werden (www. defeat-corona.de). Für die Intervention werden für die erste Interventionsgruppe über einen Zeitraum von zwölf Wochen jeweils zwei live-online Behandlungen von speziell ausgebildeten ErgotherapeutInnen (ErgotherapeutInnen der Praxis Timm-Cook Hannover) angeboten. Die zweite Interventionsgruppe erhält über einen Zeitraum von zwölf Wochen Zugang zu wöchentlich zwei, nicht individualisierten, on-demand abrufbaren Video-Ergotherapie-Modulen (aufgenommen mit SchauspielpatientInnen und TherapeutInnen der Praxis Timm-Cook Hannover). Die Inhalte der live-online Ergotherapie und der Videomodule enthalten eine identische Struktur und folgen einem speziell für diese Studie entwickelten Therapiemanual zur digitalen Anwendung.

Die ergotherapeutische Vorgehensweise ist darauf ausgerichtet, Long COVID-assoziierte, kognitive Einschränkungen wie Brain Fog, Fatigue und/oder durch Konzentrationsstörungen verursachte Handlungsprobleme im Alltag der PatientInnen zu behandeln.

4. Nicht verblindete randomisierte kontrollierte Pilotstudie

4.1 Charakterisierung der TeilnehmerInnen

An der Studie können Long COVID-PatientInnen (n=80 Erwachsene im Alter von 30-50 Jahren, n=80 Jugendliche im Alter von 16-18 Jahren) teilnehmen, bei denen nach einer PCR-bestätigten SARS- CoV-2 Infektion für mehr als vier Wochen subjektiv wahrgenommene, kognitive Symptome wie Brain Fog, Konzentrationsstörungen und Fatigue persistieren, die zu Einschränkungen in produktiven Lebensbereichen wie z.B. Schule, Ausbildung sowie Berufsleben führen.

4.1.1 Einschlusskriterien

- Erwachsene (weiblich, männlich, divers) im Alter ab 30 bis einschließlich 50 Jahren,
- Jugendliche (weiblich, männlich, divers) im Alter ab 16 bis einschließlich 18 Jahren,
- kognitive Symptome, die zu einer Long COVID Erkrankung passen und mindestens vier Wochen nach einer PCR-bestätigten Infektion mit SARS-CoV-2 persistieren,
- Zugang zu einem technischen Medium (z.B. PC, Tablet, Smartphone mit Internetzugang), mit dem an einer der digitalen Interventionsvarianten teilgenommen werden kann,
- Einwilligung der/des Teilnehmenden bzw. der/des Erziehungsberechtigten.

4.1.2 Ausschlusskriterien

- Alter unter 16 Jahre, zwischen 19-29 Jahre und über 51 Jahre,
- diagnostizierte kognitive Einschränkungen *vor* Infektion mit SARS-CoV2,
- vorangegangene SARS-CoV2 wurde nicht mittels PCR-Test bestätigt,
- kein Zugang zu PC, Tablet, Smartphone mit Internetzugang,
- kein Einverständnis zur Teilnahme.

4.2 Rekrutierung

Insgesamt sollen n=160 TeilnehmerInnen (n=80 Jugendliche, n=80 Erwachsene) für diese Studie rekrutiert werden. Das Rekrutierungsvorgehen erfolgt sukzessive über einen geplanten Zeitraum von etwa drei Monaten.

n=80 Erwachsene (weiblich, männlich, divers) im Alter ab 30 bis einschließlich 50 Jahren sollen über die bereits etablierte Online-Plattform der DEFEAT-Corona Studie (Studienregister- Nummer DRKS00026007, Ethikvotum 9948_BO_K_2021) rekrutiert werden. Als Basis der sukzessiven Rekrutierungsstrategie dienen hier Fragebogenangaben zu kognitiven Einschränkungen, deren Erhebung bereits im Rahmen der DEFEAT Studie genehmigt wurde. Wenn Probandinnen die Teilnahmekriterien erfüllen, werden diese gefragt, ob sie an der Studie teilnehmen möchten.

Zusätzlich sollen n=80 Jugendliche (männlich, weiblich, divers) im Alter ab 16 bis einschließlich 18 Jahren rekrutiert werden. Um eine Rekrutierung analog zu den erwachsenen ProbandInnen zu ermöglichen, wird ein Ammendement zur Corona-DEFEAT Studie eingereicht (siehe parallel eingereichtes Amendement mit selbem Abgabedatum wie dieses Studienprotokoll). Online wird hier nicht nur das Einverständnis der/des Jugendlichen selbst, sondern auch das Einverständnis von mindestens einem Sorgeberechtigten eingeholt.

Um die Aufmerksamkeit auf unsere Studie zu erhöhen, sollen neben der Bekanntgabe über das Corona-DEFEAT-Portal Flyer bei niedergelassenen KinderärtztInnen (oder HausärtztInnen) und in den Long COVID-Ambulanzen der Medizinischen Hochschule Hannover (MHH) und Partnerkliniken ausgelegt werden. Information über die Studie erfolgt ebenfalls über die Website der MHH.

4.3 Aufklärung und Einwilligung

Information und Aufklärung über ein Screening zur Studie erfolgt im Rahmen des bereits genehmigten Studienvorhabens (DEFEAT, Ethikvotum 9948_BO_K_2021) in einem ersten Schritt digital ("Aufklärung und Einwilligung in prospektive Beobachtungsstudie"). Kommen Teilnehmende für die Interventionsstudie infrage, werden sie darüber aufgeklärt was Ziel und Inhalt der Studie ist (siehe Anhang mit Aufklärungen). StudienteilnehmerInnen werden vor Unterzeichnung ihres Einverständnisses (bei minderjährigen nach Einverständniserteilung eines Erziehungsberechtigten) zur Studienteilnahme über Ziel, Zweck und das Vorgehen der Studie und ihrer verschiedenen Zweige informiert. Teilnehmende werden darauf hingewiesen, dass die Studie Pilot-Character hat, die Behandlung erfolglos sein kann und dass die Zustimmung zur Teilnahme jederzeit widerrufen werden kann. Diese Aufklärung erfolgt in einem persönlichen Gespräch (onsite oder fernmündlich). Die Dokumente zur Aufklärung für Erwachsene und Jugendliche (Anhänge #1-2 dieses Studienprotokolls) sowie die Einverständniserklärungen (Anhänge #3-4 dieses Studienprotokolls) finden sich im Supplement dieses Antrags.

4.4 Teilnahme und Widerruf

Die Studienteilnahme ist ausdrücklich freiwillig und kann zu jedem Zeitpunkt ohne Angabe von Gründen und ohne Konsequenzen abgebrochen werden. Dies kann durch Nichtausfüllen der digitalen Erhebungsinstrumente, dem Fernbleiben der Therapie-Sessions oder der Live-online-Erhebungen oder durch schriftliche oder fernmündliche Mitteilung erfolgen. Unter Nennung des individuell zugeordneten Pseudonyms können TeilnehmerInnen jederzeit die Vernichtung bereits erhobener Daten und Informationen erwirken.

4.5 Studienablauf – Vorgehen und Durchführung

4.5.1 *Eingangstestungen (t0)*

Studienteilnehmende erhalten nach Einwilligung in die Studienteilnahme eine Einladung zur Eingangstestung (t0), die den Status der subjektiven und objektiven kognitiven und Alltags-Einschränkungen, die im Zusammenhang mit Long COVID stehen, erheben soll. Hierzu werden bei allen Teilnehmenden folgende Instrumente zur Messung der Effektivität der Intervention eingesetzt:

1. COPM (Canadian Occupational Performance Measure) bezogen auf Produktivität,
2. WIT-2 zur Testung von Merkfähigkeit und kognitiver Arbeitseffizienz,
3. IMET-Index zur Messung von Einschränkungen der Teilhabe,
4. NeuroQual zur Erhebung der kognitiven Leistungsfähigkeit und von Fatigue-Symptomen,

Alle Testungen werden im Anhang genau beschrieben (siehe Anhänge #5-8 dieses Studienprotokolls). Zusätzlich werden zum Zeitpunkt t0 soziodemographische und medizinische Angaben erhoben (Alter, Geschlecht, Schul-/Ausbildungsform, Beruf, Zeitpunkt und Dauer der SARS-CoV-2 Infektion, Schwere der SARS-CoV-2 Infektion). Folgende Analysen sollen zu t0 durchgeführt werden:

Die Erhebung aller Analysen finden online statt und wird durch qualifiziertes Fachpersonal durchgeführt (ÄrztInnen, ErgotherapeutInnen oder MitarbeiterInnen, die von einem Psychologen (Prof. Dr. Christoph Berg, FOM Hochschule, Hamburg) geschult und supervidiert werden). Die TesterInnen sind weder BehandlerInnen während der Intervention, noch wissen Sie, welcher Gruppe die Teilnehmenden zugeordnet werden. Das COPM wird innerhalb eines 30-minütigen online-Termins durchgeführt. Die WIT-2-Testung in den Bereichen Merkfähigkeit und kognitive Arbeitseffizienz beanspruchen etwa 35 Minuten. Die Erhebung folgender Testungen finden online statt und werden durch die ProbandInnen selbstständig durchgeführt: IMET, NEuroQual, EQ-5D-5L.Die Gesamttestdauer dieser Testungen beträgt je nach kognitivem Zustand jeweils zwischen 10 und 20

Minuten. Sie soll in einer Session (ggf. zwei Sessions bei zu großer Erschöpfung der Teilnehmenden) nach der Randomisierung zu t0 und nach der letzten Therapieeinheit zu t1 sowie zu einem online vereinbarten Termin zu Zeitpunkt t2 stattfinden.

4.5.2 Randomisierung

Nach der Eingangstestung (t0) werden die Teilnehmenden drei verschiedenen Gruppen (live-online Ergotherapie: n=20 Erwachsene, n=20 Jugendliche, Videoergotherapie: n=20 Erwachsene, n=20 Jugendliche, keine Intervention: n=40 Erwachsene, n=40 Jugendliche) zugeordnet. Die Randomisierung erfolgt unter Einbezug einschlägiger digitaler Tools (z.B. Randomizer: A Randomization (Randomisation) Service for Multicenter Clinical Trials). Die n=160 Teilnehmenden werden in folgende Studienzweige randomisiert:

1. Gruppe A: n=20 Jugendliche und n=20 Erwachsene erhalten über einen Zeitraum von zwölf Wochen zweimal wöchentlich live online Ergotherapie, die jeweils 30 Minuten dauert. Diese wird von speziell ausgebildeten ErgotherapeutInnen durchgeführt.
2. Gruppe B: n=20 Jugendliche und n=20 Erwachsene erhalten über einen Zeitraum von zwölf Wochen Zugang zu nicht-individualisierten ergotherapeutischen Videomodulen (Video-Online Ergotherapie), jeweils zwei Videos pro Woche zur Selbstanwendung.
3. Gruppe C: n=40 Jugendliche und n=40 Erwachsene erhalten über einen Zeitraum von zwölf Wochen keine Intervention und bilden somit die Vergleichsgruppe.

4.5.3 Kontrolltestungen (t1 und t2)

Unmittelbar nach der zwölfwöchigen Intervention (t1), sowie zu einem weiteren Zeitpunkt (24 Wochen nach Einschluss (t2)) sollen erneut Messungen mit den identischen Instrumenten der Eingangsmessung (t0) stattfinden.

4.5.4 Datenauswertung der nicht verblindeten, randomisiert-kontrollierten Pilotstudie

Die Evaluation der Pilotstudie soll mit Hilfe eines **generalized linear mixed-effects model** (GLME; Deutsch: verallgemeinerte, lineare, gemischte Modelle) einer Klasse von Regressionsmodellen, durchgeführt werden. Dieses Modell wird angewandt, wenn der Einsatz der Kovariablen, die zur Bewertung einer Intervention analysiert werden, nicht zwingend normalverteilt ist und es Daten mit mehr als einer Quelle für zufällige Variabilität gibt. In diesem Fall zeigen sich bei geplanten, wiederholten Messungen im Laufe der Zeit wahrscheinliche Variation bei einer ProbandIn und zwischen den ProbandInnen. Das Modell soll die Beziehungen aufzeigen, zwischen

1. messbaren PatientInnen- und TherapeutInnen-Merkmalen und Remissionswahrscheinlichkeit,
2. zufälliger Variabilität in PatientInnengruppen und in individuellen Remissionsergebnissen und
3. zwischen den verschiedenen TherapeutInnen und jeweiligen Remissionswahrscheinlichkeiten.

Das GLME soll helfen, eine fundierte Entscheidung darüber zu treffen, ob die individuellen Unterschiede der Krankheitsverläufe nach Intervention mit der digital vermittelten Ergotherapie oder mit einzelnen Faktoren der Intervention korrelieren.

4.5.6 *Prozessevaluation mittels Realist Evaluation*

Zusätzlich soll eine Prozessevaluation mittels Realist Evaluation durchgeführt werden, bei der die Evaluation von Wirksamkeit und Machbarkeit der Pilotstudie fokussiert wird. Nach Bastian et al. (2009) stehen bei der Realist-Evaluation keine linearen Ursache-Wirkungs-Aussagen, sondern das

Zusammenspiel von Kontexten durch soziale Interaktionen im Vordergrund, welche die Intervention in Anspruch nehmen. In Anlehnung an diese Methode soll im Rahmen dieser Studie erhoben werden, welche Wirkungen und Einflüsse die digitale ergotherapeutische Intervention (live online Ergotherapie-Einheiten bzw. on-demand Videos) auf den Gesundheitszustand der StudienteilnehmerInnen hat, als auch die Machbarkeit bzw. Anwendbarkeit beider Interventionszweige aus Sicht der NutzerInnen.

Hierzu soll nach jedem Interventionsmodul eine Evaluation der Inhalte durch die StudienteilnehmerInnen beider Interventionsgruppen erfolgen. Diese werden den Teilnehmenden über die online-Plattform mit dem Tool SoSci Survey ein Onlinefragebogen (Anhang #9) am Ende jedes Moduls bereitgestellt.

Nach Abschluss der Intervention werden zusätzlich qualitative ExpertInneninterviews mit den Ergotherapeutinnen und einigen Studienteilnnehmerinnen durchgeführt um die Perspektive der behandelnden ErgotherapeutInnen und StudienteilnehmerInnen hinsichtlich der Anwendbarkeit, Akzeptanz, Zeitaufwand sowie ihrem Umgang mit den digitalen Interventionsformen zu erheben. Seitens der StudienteilnehmerInnen werden zudem auch deren Wahrnehmung von Auswirkungen der Intervention auf die Konzentration und Verbesserung der alltäglichen Lebensführung abgefragt. Darüber hinaus sollen Angaben zur Therapietreue und Gründe für Dropouts etc. ermittelt werden.

Ziel dieses Vorgehens ist es, die Interventionsformen hinsichtlich ihrer Durchführbarkeit, Angemessenheit und Handhabbarkeit zu ermitteln. Hierzu sollen alle behandelnden ErgotherapeutInnen befragt werden. StudienteilnehmerInnen werden nach dem Zufallsprinzip ausgewählt (zB über https://www.randomizer.at/). Insgesamt 16 Studenteilnehmende (n=16) sollen befragt werden: Jugendliche (n=8, n=4 davon weiblich), zu gleichen Anteilen aus beiden Interventionsgruppen; Erwachsene (n=8, n=4 davon weiblich) zu gleichen Anteilen aus beiden Interventionsgruppen.

4.6 Pseudonymisierung und Datenschutz

Der Datenschutz wird gemäß rechtlichen Datenschutzbestimmungen umgesetzt. Die ProbandInnen werden über alle wesentlichen datenschutzrechtlichen Aspekte aufgeklärt und geben nach der Aufklärung ihr schriftliches Einverständnis.

Die in der vorliegenden Untersuchung erhobenen Daten gliedern sich in vier Datensätze:

1. Personenbezogene Daten (Name, Emailadresse)
2. Soziodemographische Daten, klinische Charakterisierung (Angaben zu Alter, Geschlecht, Migrationshintergrund, Gesundheitsstatus, Symptome etc.) sowie Einstellung gegenüber Impfung und Erfahrungen mit COVID-19 Impfung
3. Testresultate (Scores in kognitiven Testungen etc.)

und hiervon getrennt:

4. Qualitative Daten (Gesprächsnotizen)

Um sie vor Missbrauch zu schützen, fließen Daten aus den ersten drei Bereichen in zwei unabhängige Datenbanken ein, die getrennt voneinander gespeichert werden. Personenbezogene Daten (Datensatz 1), werden physikalisch getrennt von sämtlichen weiter erhobenen Daten (Datensatz 2 und 3), verschlüsselt gespeichert.

Die personenbezogenen Daten und Einwilligungserklärungen werden ausschließlich im jeweils einschließenden Studienzentrum gespeichert. Die Daten der Online-Umfrage werden über das von

der Medizinischen Hochschule zur Verfügung gestellte Umfrageprogramm SoSci Survey erhoben. Dies zeichnet standardmäßig keine IP-Adressen auf, die Daten werden auf dem Server des Rechenzentrums der MHH gemäß den datenschutzrechtlichen Vorschriften, insbesondere der DSGVO, gespeichert und verarbeitet. Die Daten des qualitativen Studienteils werden auf Servern der Universitätsmedizin Göttingen gespeichert. 10 Jahre nach Ablauf der Studie werden die Daten unwiederbringlich vernichtet. In Papierform erhobene Einwilligungsbögen werden in zugriffssicher verschließbaren Aktenschränken aufbewahrt. Nur die jeweiligen StudienleiterInnen haben Zugriff. Das wissenschaftliche und BehandlerInnenteam hat keinen Zugriff auf die personenbezogenen Daten. Mit Hilfe einer Zeichenkombination (5-stellige Zahlen/Buchstabenkombination) werden die Datensätze 2 und 3 pseudonymisiert (pseudonymisierte ID, PID), so dass ein Rückschluss auf die Identität der Studienteilnehmer nicht möglich ist. Die Verarbeitung und Einlagerung der Daten sowie die gesamte Datenverarbeitung aller nicht personenbezogenen Daten erfolgen mittels PID.

Ein PID-Schlüssel erlaubt die Zuordnung von personenbezogenen Daten (Datensatz 1) zu pseudonymisierten Daten (Datensatz 2 und 3). Über diesen Schlüssel verfügt ausschließlich das jeweilige Studienzentrum. Dieser Schlüssel wird bei Einschluss in die Studie durch das rekrutierende Studienzentrum erstellt und an den Datentreuhänder weitergegeben. Diese Aufgabe liegt bei einer festgelegten Person aus dem Studienteam, welche voraussichtlich die Forschungsgruppenleiterin ist. Keine anderen Personen können darauf zugreifen. Das Studienzentrum verfügt nur über den PID-Schlüssel und hat keinen direkten Zugang zu den Datensätzen 2 und 3.

Die Re-Identifizierung, d.h. die Verknüpfung von persönlichen Daten und pseudonymisierten Datensätzen, kann in bestimmten Fällen notwendig sein: Die StudienteilnehmerInnen ordnen die Vernichtung von Daten an; die StudienteilnehmerInnen haben einer erneuten Kontaktierung durch das Studienteam zugestimmt und es werden Nachuntersuchungen und/oder die erneute Gewinnung von Daten notwendig. Es ist Gefahr im Verzug. In diesen Ausnahmefällen bzw. aus ähnlichen oder entsprechenden Gründen ist ein Zugriff auf persönliche Daten vorgesehen. Der Zugriff kann ausschließlich von der Projektleitung initiiert werden. Hierzu müssen folgende Voraussetzungen erfüllt sein: Es muss das Einverständnis der/des ProbandIn zur wiederholten Kontaktaufnahme durch das Studienteam vorliegen. Die Ethik-Kommission der Medizinischen Hochschule Hannover muss der Aufhebung der Pseudonymisierung zustimmen. Nur unter diesen Bedingungen erfolgt eine Herausgabe des PID-Schlüssels, welche die Zuordnung von personenbezogenen Daten und PID ermöglicht. Dieses Konzept ermöglicht es, dass keine MitarbeiterIn gleichzeitig Zugang zum Klarnamen und Studienergebnissen erlangen kann.

Bei dem gewählten Vorgehen wird auf die Erhebung von personenbezogenen Daten (wie etwa Geburtsdatum, Adresse o.ä.) weitgehend verzichtet. Durch die physische Trennung von Aufklärungsunterlagen und Fragebögen / Proben wird eine größtmögliche Sicherheit geschaffen. Selbst im unwahrscheinlichen Fall eines Datenverlusts ist eine retrograde Identifikation der ProbandInnen unwahrscheinlich. StudienmitarbeiterInnen sind auf Einhaltung der geltenden Datenschutz- und Datensicherheitsbestimmungen verpflichtet.

Qualitative Daten (3) werden ausschließlich bei der Universitätsmedizin Göttingen prozessiert, andere ProjektmitarbeiterInnen haben darauf keinen Zugriff. Nach Transkription von Gesprächsnotizen werden Transkripte pseudonymisiert, andere Hinweise (Wohnort, Namen von ÄrztInnen o.ä.) die eine retrograde Identifikation ermöglichen, werden getilgt (faktische Anonymisierung). Audiorohdaten / Videodaten werden anschließend vernichtet. Transkripte werden auf digitalen Datenträgern mit Passwortschutz in einem verschließbaren Aktenschrank verwahrt. Transkripte werden, wenn der Proband keiner Nutzung für weitere Forschungsprojekte der faktisch anonymisierten Daten zustimmt, nach 10 Jahren gelöscht.

5. Liste der Anhänge

1. Aufklärung Jugendliche 16.-18. Lebensjahr (inklusive Elternaufklärung)
2. Aufklärung Erwachsene 30.-50. Lebensjahr
3. Einverständniserklärung Jugendliche 16.-18. Lebensjahr (inkl. Elterneinverständniserklärung)
4. Einverständniserklärung Erwachsene 30.-50. Lebensjahr
5. Angaben zum Testinstrument COPM, (Canadian Occupational Performance Measure)
6. Angaben zum Testinstrument Wit2
7. Angaben zum Testinstrument IMET zur Messung von Einschränkungen der Teilhabe
8. Angaben zum Testinstrument NeuroQual zur kognitiven Leistungsfähigkeit und Fatigue
9. Fragen zur Prozessevaluation

6. Literaturverzeichnis

Al-Aly, Z., Bowe, B. & Xie, Y. Long COVID after breakthrough SARS-CoV-2 infection. *Nature Medicine* (2022). https://doi.org:10.1038/s41591-022-01840-0

AOTA. (2021). *Information Pertaining to Occupational Therapy in the Era of Coronavirus (COVID-19).* Retrieved 04 28, 2022, from American Occupational Therapy Association: https://www.aota.org/Practice/ Health Wellness/COVID19.aspx.

Barkel, C., Lap, C. R., Buddingh, E. P., van Houten, M. A., van der Sande, L., Langereis, E., & Terheggen-Largro, S. (2021). Pediatric long-Covid: An overlooked phenomenon? *Pediatric pulmonology*, 2495-2502. doi:10.1002/ppul.25521

Beli, S., Babi, B., Price, I., Cattaneo, D., Masocco, F., Zaccaria, S., & Spruit, M. A. (2020). Low physical functioning and impaired performance of activities of daily life in COVID 19 patients who survived hospitalisation. *Eur.Respir J, 15*(56). doi:doi:10.1183/13993003.02096-2020

DVE, D. V. (2018). *Kompetenzprofil Ergotherapie.* Retrieved 04 28, 2022, from DVE: https://dve.info/resources/pdf/ergotherapie/kompetenzprofil-ergotherapie/3633-2019-kompetenzprofil/file

Hugon, J., Msika, E., Queneau, M., Farid, K., & Paquet, C. (2021). Long COVID: cognitive vomplaints (brain fog)and dysfunction of the cinglate cortes . *Journal of neurology*, 1-3.

Kassenärztliche Bundesvereinigung. (2021, 07 14). Retrieved from https://www.kbv.de/html/115_52751.php

Kataria, S., & Ravindran, V. (2018). Digital health: a new dimension in rheumatology patient care. *Rheumatol Int., 38*(11), 1949-1957. doi:10.1007/s00296-018-4037-x.

Kos, D., Duportail, M., Meirte, J., Meeus, M., D'hooghe, M., & Nagels, G. (2016). The effectiveness of a self-management occupational therapy intervention on activity performance in individuals with multiple sclerosis-related fatigue: a randomized-controlled trial. *International journal of rehabilitation research*.

Koczulla, A. R. *et al.* S1-Leitlinie Post-COVID /Long COVID. *Pneumologie* **75**, 869-900, doi:10.1055/a-1551-9734 (2021).

le Granse, M., van Hartingsveldt, M., & Kinébanian , A. (2017). *Grondslagen van de Ergotherapie.* Houten: Bohn Stafleu van Loghun.

Lopez-Leon, S., Wegman-Ostrosky, T., Perelman, C., Sepulveda, R., Rebolledo, P., Cuapio, A., & Villapol, S. (2021). More than 50 Long-term effects ov COVID-19: a systematic review and meta-analysis. *medRciv*. doi:10.1101/2021.01.27.21250617

Margetis, J. L., Wilcox, J., Thompson, C., & Mannion, N. (2021). Occupational therapy: Essential to critical care rehabilitation. *American Journal of occupational therapy, 75*(2), 7502170010p1-7502170010p5. doi:10.5014/ajot.2021.048827

Miller, F., Nguyen, V., Navaratnam, A., Shrotri, M., Kovar, J., Hayward, A., & Hardelid, P. (2021). Prevalence of persistent symptoms in children during the COVID-19 pandemic: evidence from a household cohort study in England and Wales. *medRxiv*. doi:10.1101/2021.05.28.21257602

NICE. (2022). *Rapid guideline: managing the long term effects of COVID-19.* Retrieved from https://www.nice.org.uk/guidance/ng188/resources/covid19-rapid-guideline-managing-the-longterm-effects-of-covid19-pdf-51035515742

Nalbandian, A. (2021) Post-acute COVID-19 syndrome. Nature Medicine. 27,601-615 doi:https://doi.org:10.1038/s41591-021-01283-z

Nittas, V., Gao, M., West, E. A., Ballouz, T., Menges, D., Wulf Hanson, S., & Puhan, M. A. (2022). Long Covid through a Public Health Lens: An Umbrella Review. *Public HEalth Reviews*. doi:10.3389/phrs.2022.1604501

Peine, A., Paffenholz, P., Martin, L., Dohmen, S., Marx, G., & Loosen, S. H. (2020). Telemedicine in Germany During the COVID-19 Pandemic: Multi-Professional National Survey. *J Med Internet Res, 22*(8), e19745. doi:10.2196/19745

Townsend, L., Dyer, A. H., & Jones, K. (2020). Persistent fatigue following SARS-CoV-2 Infection is common and independent of severity of initial infection. *Plos One, 15*(11), e0240784.

Walzer, S. (2022). Digital Healthcare in Germany, Contributions to Economics. Springer Nature. doi:https://doi.org/10.1007/978-3-030-94025-6_2

Wong-Chew,R.(2022). Symptom cluster analysis of long COVID-19 in patients discharged from the Temporary COVID-19 Hospital in Mexico City. Therapeutic Advances in Infectious Disesase doi:https://doi.org/10.1177/20499361211069264

7. Anhänge

Anhang 1: Aufklärung Jugendliche ab 16. Lebensjahr (inklusive Elternaufklärung)

Studienleitung
PD Dr. Alexandra Dopfer-Jablonka

Zentrale Kontaktstelle
PD Dr. med. Alexandra Dopfer-Jablonka
Klinik für Immunologie und Rheumatologie - OE 6830
Medizinische Hochschule Hannover
Carl Neuberg Straße 1
30625 Hannover
Email: Jablonka.Alexandra@mh-hannover.de
Tel: +49 511 532 3014

Aufklärung für Jugendliche ab 16 Jahren zur Studie: „ErgoLoCo" – online Ergotherapie bei Long COVID

Liebe Teilnehmende Jugendliche,

liebe Eltern, liebe Erziehungsberechtigte,

wir laden Dich/ Sie und Ihr Kind ein, an der Studie zu unserem Modellprojekt „**ErgoLoCo"** zu **online Ergotherapie** teilzunehmen. In der Studie versuchen wir, Long COVID bei Jugendlichen und Erwachsenen besser zu verstehen und besser behandeln zu können.

Im Folgenden möchten wir Sie/Dich bzw. Ihr Kind über die Ziele und den Verlauf dieser Untersuchung informieren. Weiterhin möchten wir Dir/ Ihnen erklären, warum die Teilnahme wichtig ist. Wir bitten Sie/ Dich/ Ihr Kind die vorliegenden Informationen sorgfältig durchzulesen und anschließend zu entscheiden, ob eine Studienteilnahme gewünscht ist oder nicht.

Die Teilnahme an dieser Studie ist freiwillig. Eine Teilnahme erfolgt nur, wenn Du/ Sie dazu Ihre Einwilligung geben. Sofern die Teilnahme jetzt oder später verweigert oder widerrufen wird, entstehen Dir/ Ihnen/ Ihrem Kind keine Nachteile.

Da Du/Sie sich für die Studie interessieren, liegen Dir/ Ihnen wahrscheinlich schon eine Reihe von Informationen zu der geplanten Studie vor. Der nachfolgende Text soll noch einmal über die wichtigsten Aspekte der Studie – insbesondere über die Ziele und den Ablauf – informieren. Lies Dir/Lesen Sie sich deshalb die Teilnahmeinformation bitte sorgfältig und gewissenhaft durch. Anschließend wird eine StudienmitarbeiterIn bei einem Online-Termin mit Ihnen ein Aufklärungsgespräch führen. Bitte zögere nicht/ zögern Sie nicht, alle Punkte anzusprechen, die Ihnen unklar sind. Du wirst/ Sie werden ausreichend Bedenkzeit erhalten, um über die Teilnahme von Dir

und/oder Ihres Kindes zu entscheiden. Für weitere Informationen kannst Du dich/ können Sie sich jederzeit an unser Studienteam wenden (Kontaktdaten siehe oben).

Teil I: Aufklärung über die Studie

1. Welche Ziele hat die Studie?

Mit der Studie soll gezeigt werden, ob wir Konzentrationsprobleme, an denen Jugendliche und Erwachsene mit Long COVID leiden, verbessern können, wenn wir den Patientinnen Online Ergotherapie (eine Form von Bewegungs-/ Beschäftigungsanleitung), die in Videos oder Live-online Tutorials vermittelt wird, zur Verfügung stellen.

Im Moment gibt es sehr viele PatientInnen mit Konzentrationsproblemen nach COVID-19. Leider haben wir noch keine guten Therapiemöglichkeiten. Wir wollen testen, ob online vermittelte Ergotherapie Konzentrationsprobleme bei Long COVID verbessert. Dazu sollen die Hälfte aller TeilnehmerInnen online Ergotherapie-Sessions erhalten, die andere Hälfte erhält als Kontrollgruppe keine Intervention. In welche Gruppe Du/ Sie/ Ihr Kind fällst/ fallen, wird zufällig entschieden und nach den ersten Tests mitgeteilt.

2. Welche Methoden werden verwendet und wie wird die Studie ablaufen?

Wenn sich aus den Online-Tests oder einer Untersuchung durch eine betreuende ÄrztIn der begründete Verdacht ergibt, dass es sich um Konzentrationsprobleme wegen Long COVID handeln könnte, wirst Du/ werden Sie und Deine/ Ihre Eltern über die Studie aufgeklärt und um Zustimmung zur Teilnahme gebeten.

Das Studienteam wird Ihnen/ Dir und Ihren/ Deinen Eltern diese Aufklärungsunterlagen geben und in einem live-online Gespräch alle Studienmaßnahmen erklären.

Wenn Du/ Sie und Ihre/ Deine Eltern mit der Teilnahme an der Studie einverstanden sind, bekommen Sie/bekommst Du/ bekommt Ihr Kind eine Studien-Nummer. Alle Daten für die Studie werden dann nur mehr unter dieser Studiennummer gespeichert (pseudonymisiert).

Wenn zur Teilnahme zugestimmt wird, vereinbaren wir zwei Online-Termine zu Testung von Ihrer/ Deiner Konzentrations- und Merkfähigkeit, Lebensqualität und Teilhabe (Ihres Kindes) im Alltag. Dadurch können wir einschätzen, wie schlimm die Einschränkungen durch Long COVID im Alltag sind und haben einen Ausgangswert, um abschätzen zu können, ob sich durch die online Ergotherapie etwas verbessert. Für die Tests und die spätere Ergotherapie brauchen Sie/ brauchst Du/ braucht Ihr Kind ein online-fähiges Gerät wie einen PC oder ein Tablet mit Mikrofon und Kamera. Die Tests und auch die spätere Therapie werden ausschließlich von geschulten, zur Verschwiegenheit verpflichteten MitarbeiterInnen in einem geschützten digitalen Raum durchgeführt. Die Tests beim ersten Termin dauern etwa 30 Minuten.

Anschließend findet eine zufällige Zuordnung in die verschiedenen Studienarme (Randomisierung) statt. Zufällig wird entschieden, ob Du/ Sie/ Ihr Kind in eine Interventions- oder in eine Kontrollgruppe kommt.

Während die Kontrollgruppe (50% aller TeilnehmerInnen) keine Behandlung erhält und nach zwölf und 24 Wochen erneut auf die Konzentrationsfähigkeit und Lebensqualität hin getestet wird (wieder mittels online-Termin, Dauer ca. 30 Minuten), erhalten die Interventionsgruppen (50% der

Teilnehmenden) von Woche null bis zwölf jeweils zweimal pro Woche halbstündige, digitale Ergotherapie-Sessions. Dabei erhalten 25% aller Teilnehmenden live online Ergotherapie, treffen sich also online mit ErgotherapeutInnen (Praxis Timm-Cook, Hannover) und werden in Techniken geschult, die ihnen den Umgang mit den Konzentrationsproblemen im Alltag erleichtern.

Die andere Hälfte der Teilnehmenden in der Interventionsgruppe (25% aller Menschen, die an der ersten Testung teilgenommen haben), erhalten digitale Ergotherapie mittels zuvor aufgenommenen, speziell auf Long COVID abgestimmten Ergotherapievideos (ebenfalls zweimal eine halbe Stunde pro Woche. Die Videos können „on-demand" abgerufen werden.

Beide Interventionsgruppen erhalten auch Anleitungen zum regelmäßigen Training im Alltag, die helfen sollen, besser mit den Einschränkungen durch Long COVID zurecht zu kommen.

Zusätzlich zur Testung der kognitiven Fähigkeiten und Lebensqualität werden wir Sie/ Dich/ Ihr Kind bitten, uns die Sicht zur Diagnostik, Behandlung sowie zur körperlichen und psychischen Gesundheit mitzuteilen. Wenn Sie/Du und Ihre/Deine Eltern einverstanden sind, werden wir dazu Fragebögen zur Beantwortung in pseudonymisierter Form geben (online oder wenn gewünscht als Papierfragebogen).

Alle Daten werden pseudonymisiert, also ohne direkten Rückschluss auf die teilnehmende Person ausgewertet. Durch die Auswertung der Daten wollen wir erreichen, dass Long COVID bei Jugendlichen und Erwachsenen in Zukunft besser behandelt werden kann.

3. Welche Risiken entstehen bei einer Teilnahme für mich?

Risiken könnten sich theoretisch aus dem Datenschutz ergeben. Das wird in Teil 2 dieser Aufklärung genau erklärt.

4. Wann darf ich nicht an der Studie teilnehmen?

Von der Studienteilnahme ausgeschlossen sind:

- Alter unter 16 Jahre, zwischen 19-29 Jahre und über 51 Jahre,
- diagnostizierte kognitive Einschränkungen *vor* Infektion mit SARS-CoV2,
- die vorangegangene SARS-CoV2 wurde nicht mittels PCR- Test bestätigt,
- kein Zugang zu PC, Tablet, Smartphone mit Internetzugang,
- Kein Einverständnis zur Teilnahme.

5. Kann ich die Studie vorzeitig beenden?

Die Teilnahme an der Studie kann jederzeit ohne Angabe von Gründen beendet werden. Die Beendigung der Teilnahme hat keinerlei negative Effekte für Sie/ Dich/ Ihr Kind.

6. Welcher persönliche Nutzen entsteht für mich und bekomme ich eine Aufwandsentschädigung?

Durch die Teilnahme haben Sie/ hast Du/ hat Ihr Kind die Chance, an einem neuartigen Therapieversuch für Long COVID teilzunehmen. Wir nehmen Long COVID ernst und hoffen, dass die Symptome durch eine strukturierte Online-Intervention gebessert werden können. Aber es muss allen Teilnehmenden klar sein, dass die Studie eine Pilot-Studie mit experimentellem Charakter ist. Das heißt, dass wir nicht sagen können, ob sich eine Besserung der Symptome von Long COVID durch die digitale Intervention ergibt. Wir können auch nicht sagen, inwiefern die Therapieart von den

PatientInnen angenommen wird. Für den einzelnen Studienteilnehmer könnte die Teilnahme also möglicherweise von Vorteil sein, das übergeordnete Ziel der Studie ist es aber, Therapiemöglichkeiten bei Long COVID im Allgemeinen besser zu verstehen und die Versorgung für alle Jugendlichen (junge Erwachsene) und Erwachsenen mit dieser Erkrankung zu verbessern. Ob davon bereits Studienteilnehmer selbst profitieren, kann nicht vorhergesagt werden. Eine Aufwandsentschädigung ist nicht vorgesehen.

7. Was passiert, wenn sich durch die Untersuchung Zufallsbefunde ergeben?

Da es im Rahmen der Studie zu Ergebnissen bzw. Erkenntnissen kommen kann, die auf eine andere Erkrankung hindeuten, welche nicht Teil der Studie ist, bitten wir Sie/Dich und Ihre/Deine Eltern uns vor Beginn der Studie mitzuteilen, ob Sie auf diese Befunde aufmerksam gemacht werden wollen (siehe Einwilligungserklärung). Je nach Befund ergeben sich daraus weitere Empfehlungen hinsichtlich Therapie oder weiterer Untersuchungen.

8. Gibt es eine Studien-/Unfall-/Wegeversicherung?

Da die Begleitstudie nur online stattfindet, wurde keine Studien-/ Unfall- oder Wegeversicherung abgeschlossen.

Teil II: Datenschutzerklärung

II. 1. Was geschieht mit meinen Daten?

Zum Zweck der Durchführung der klinischen Studie werden medizinische Befunde und persönliche Informationen (wie Alter, Geschlecht, Zeitpunkt der COVID-19 Erkrankung) über Dich/ Sie/ Ihr Kind erhoben und durch StudienmitarbeiterInnen in der Studiendatenbank dokumentiert.

Die für die klinische Studie wichtigen Daten werden nur in verschlüsselter (pseudonymisierter) Form in einer passwortgeschützten, elektronischen Datenbank gespeichert. Pseudonymisiert bedeutet, dass keine Angaben von Namen bzw. Initialen verwendet werden, sondern nur ein Zahlen- oder Buchstabencode. Die Daten sind gegen unbefugten Zugriff gesichert. Nur die Studienleitung vor Ort und deren Team werden in der Lage sein, Sie/ Dich/ Ihr Kind persönlich anhand der verschlüsselten Daten zu identifizieren. Die Weitergabe der Studiendaten an die Studienzentrale erfolgt in pseudonymisierter Form. Die Weitergabe der Studiendaten an Dritte erfolgt ausschließlich in anonymisierter Form; das bedeutet, dass eine Zuordnung zu Ihnen/ Dir/ Ihrem Kind nicht mehr möglich ist.

Ihr/ Dein Name/Der Name Ihres Kindes und das Geburtsdatum werden auf der Einwilligungserklärung eingetragen, die getrennt von allen anderen Studiendaten aufbewahrt werden. Es ist möglich, dass InspektorInnen von offiziellen Überwachungsbehörden Einsicht in diese Dokumente nehmen, um die vorschriftsgemäße Durchführung der Studie zu überprüfen. InspektorInnen sind verpflichtet, Ihre (Deine/die persönlichen Daten Ihres Kindes vertraulich zu behandeln.

Die Rechtsgrundlage zur Verarbeitung der betreffenden personenbezogenen Daten ist Ihre freiwillige schriftliche Einwilligung gemäß DSGVO (nach Art. 6 Abs. 1 lit. a) DSGVO i. V. m. Art. 9 Abs. 2 lit. a) DSGVO) bei der Verarbeitung sensibler Daten. Ihre Einwilligung ist freiwillig und kann jederzeit ohne nachteilige Auswirkungen mit der Wirkung für die Zukunft widerrufen werden. Ohne Ihre Einwilligung

zur Verarbeitung und Weitergabe der betreffenden Daten in verschlüsselter Form, können Sie/ kannst Du/ kann Ihr Kind nicht an der oben genannten klinischen Studie teilnehmen. Veröffentlichungen in Fachjournalen und öffentlichen Studienregistern (z. B. clinicaltrials.gov oder EU Clinical Trials Register) oder Präsentationen von Studienergebnissen werden keinerlei Daten beinhalten, anhand derer Ihr Kind persönlich identifiziert werden kann.

Deine Daten/die Daten Ihres Kindes werden in dieser Studie primär zum oben genannten Studienzweck, nämlich der Verbesserung von Diagnose und Therapie bei Kindern und Jugendlichen mit Long COVID, verarbeitet. Jedoch kann es vorkommen, dass im Laufe der Untersuchung und Datenauswertung weitere Forschungsfragen aufkommen, die mit dem Gegenstand der hier vorliegenden Studie verwandt sind. In diesem Fall würden Deine Daten/die Daten Ihres Kindes ebenfalls für diesen Zweck verwendet werde. Sie können dem jedoch in der Einwilligung explizit widersprechen.

Deine/die von Ihrem Kind erhobenen Daten werden von der Studienzentrale bis 31.12.2023 gespeichert. Danach werden die Daten Ihres Kindes komplett anonymisiert bzw. gelöscht. Nach Anonymisierung oder Löschung ist ein Rückschluss auf Ihr Kind nicht mehr möglich. Die Verantwortung für die Datenverarbeitung im Rahmen dieser klinischen Studie liegt beim:

Prof. Georg Behrens, Klinik für Rheumatologie und Immunologie, Carl-Neuberg-Str. 1, 30625 Hannover

II. 3. Ergänzende Information gemäß Europäischer Datenschutz-Grundverordnung

Bezüglich der Daten hast Du/ haben Sie folgende Rechte, die Sie gegenüber dem Verantwortlichen geltend machen können:

Recht auf Auskunft: Sie haben das Recht auf Auskunft über die Ihr Kind betreffenden personenbezogenen Daten, die im Rahmen der klinischen Studie erhoben, verarbeitet oder ggf. an Dritte übermittelt werden (einschließlich einer kostenfreien Kopie). Auch können Sie die Überlassung eines tragbaren elektronischen Datenträgers, auf dem die Ihr Kind betreffenden Daten strukturiert und in einem gängigen Format (Office- oder PDF-Datei) gespeichert werden, oder die Übermittlung dieser Daten an einen anderen Verantwortlichen* verlangen (Artikel 15 DSGVO).

Recht auf Löschung: Sie haben das Recht auf Löschung Ihr Kind betreffender personenbezogener Daten, z. B. wenn diese Daten für den Zweck, für den sie erhoben wurden, nicht länger benötigt werden (Artikel 17 DSGVO).

Recht auf Einschränkung der Verarbeitung: Unter bestimmten Voraussetzungen haben Sie das Recht, eine Einschränkung der Verarbeitung zu verlangen, d. h. die Daten dürfen nur gespeichert, aber nicht verarbeitet werden. Dies müssen Sie beantragen (Artikel 18 DSGVO).

Recht auf Datenübertragbarkeit: Sie haben das Recht, die Ihr Kind betreffenden personen-bezogenen Daten, die Sie dem* Verantwortlichen* für die klinische Studie bereitgestellt haben, zu erhalten. Damit können Sie beantragen, dass diese Daten (strukturiert und in einem gängigen Format auf einem tragbaren elektronischen Datenträger) entweder Ihnen oder einem anderen von Ihnen benannten (weiteren) Verantwortlichen für die Datenverarbeitung im Sinne der DSGVO übermittelt werden können (Artikel 20 DSGVO).

Widerspruchsrecht: Sie haben das Recht, jederzeit gegen konkrete Entscheidungen oder Maßnahmen zur Verarbeitung der Ihr Kind betreffenden personenbezogenen Daten Widerspruch einzulegen. Eine Verarbeitung (neuer Daten) findet anschließend nicht mehr statt, es sei denn, die Verarbeitung ist gesetzlich weiterhin

gefordert – wie im Arzneimittelgesetz AMG (Artikel 21 DSGVO). Möchten Sie diese Rechte in Anspruch nehmen, wenden Sie sich bitte an Ihren* Prüfer* oder an den* Datenschutzbeauftragten* Ihres Prüfzentrums.

Einschränkungen: Wir möchten Sie an dieser Stelle darauf hinweisen, dass die aufgeführten Rechte eingeschränkt werden können, wenn diese Rechte die Verwirklichung der Forschungszwecke unmöglich machen oder ernsthaft beinträchtigen und die Beschränkung für die Erfüllung der Forschungszwecke notwendig ist (Artikel 89 DSGVO, §27 BDSG-neu). Die Rechte Ihres Kindes auf Auskunft, Datenübertragbarkeit und Berichtigung fehlerhaft verarbeiteter Daten bestehen nicht, sofern die Auskunftserteilung einen unverhältnismäßigen Aufwand erfordern würde oder technisch unmöglich ist. Ob die Rechte Ihres Kindes eingeschränkt werden können, bedarf einer konkreten Abwägung.

Sie haben das **Recht, Beschwerde bei einer Aufsichtsbehörde einzulegen,** wenn Sie der Ansicht sind, dass die Verarbeitung der Sie betreffenden personenbezogenen Daten gegen die DSGVO verstößt.

Datenschutzbeauftragter* / Datenschutz-Aufsichtsbehörde

Landesbeauftragte für den Datenschutz Niedersachsen (LfD)

Postfach 221

30002 Hannover

Tel.: 0511 120-4500, Fax: 0511 120-4599

E-Mail: poststelle@lfd.niedersachsen.de

Datenschutzbeauftragte/r der Medizinischen Hochschule Hannover

Carl-Neuberg-Str. 1

30625 Hannover

Tel.: 0511- 532-2555

E-Mail: Datenschutz@mh-hannover.de

____

(Datum, Name & Unterschrift Versuchsleiter*in bzw. Prüfarzt*in)

____

(Datum, Name & Unterschrift teilnehmende*r Jugendliche*r

____

(Datum, Name & Unterschrift Srogeberechtigte

Anhang 2: Aufklärung Erwachsene

Studienleitung
PD Dr. Alexandra Dopfer-Jablonka

Zentrale Kontaktstelle
PD Dr. med. Alexandra Dopfer-Jablonka
Klinik für Immunologie und Rheumatologie - OE 6830
Medizinische Hochschule Hannover
Carl Neuberg Straße 1
30625 Hannover
Email: Jablonka.Alexandra@mh-hannover.de
Tel: +49 511 532 3014

Aufklärung für Erwachsene zur Studie:

„ErgoLoCo“ – online Ergotherapie bei Long-COVID

Liebe Teilnehmende,

wir laden Sie ein, an der Studie zu unserem Modellprojekt „**ErgoLoCo“** zu **online Ergotherapie** teilzunehmen. In der Studie versuchen wir, Long COVID bei Jugendlichen und Erwachsenen besser zu verstehen und besser behandeln zu können.

Im Folgenden möchten wir Sie über die Ziele und den Verlauf dieser Untersuchung informieren. Weiterhin möchten wir Ihnen erklären, warum die Teilnahme wichtig ist. Wir bitten Sie die vorliegenden Informationen sorgfältig durchzulesen und anschließend zu entscheiden, ob eine Studienteilnahme gewünscht ist oder nicht.

Die Teilnahme an dieser Studie ist freiwillig. Eine Teilnahme erfolgt nur, wenn Sie dazu Ihre Einwilligung geben. Sofern die Teilnahme jetzt oder später verweigert oder widerrufen wird, entstehen Ihnen keine Nachteile.

Da Sie sich für die Studie interessieren, liegen Ihnen wahrscheinlich schon eine Reihe von Informationen zu der geplanten Studie vor. Der nachfolgende Text soll Sie über die wichtigsten Aspekte der Studie – insbesondere über die Ziele und den Ablauf – informieren. Lesen Sie sich deshalb die Teilnahmeinformation bitte sorgfältig und gewissenhaft durch. Anschließend wird eine StudienmitarbeiterIn bei einem Online-Termin mit Ihnen ein Aufklärungsgespräch führen. Bitte zögern Sie nicht, alle Punkte anzusprechen, die Ihnen unklar sind. Sie werden ausreichend Bedenkzeit erhalten, um über die Teilnahme zu entscheiden. Für weitere Informationen können Sie sich jederzeit an unser Studienteam wenden (Kontaktdaten siehe oben).

Teil I: Aufklärung über die Studie

1. Welche Ziele hat die Studie?

Mit der Studie soll gezeigt werden, ob wir Konzentrationsprobleme, an denen Jugendliche und Erwachsene mit Long COVID haben, verbessern können, wenn wir den Patientinnen Online Ergotherapie (eine Form von Bewegungs-/ Beschäftigungsanleitung), die in Videos oder Live-online Tutorials vermittelt wird, zur Verfügung stellen.

Im Moment sehen wir einen Anstieg an Fällen von schweren Alltags- und Konzentrationsproblemen nach COVID-19. Leider haben wir noch keine guten Therapiemöglichkeiten. Wir wollen testen, ob online vermittelte Ergotherapie Konzentrationsprobleme bei Long COVID verbessert. Dazu soll die Hälfte aller TeilnehmerInnen online Ergotherapie-Sessions erhalten, die andere Hälfte erhält als Kontrollgruppe keine Intervention. In welche Gruppe Siefallen, wird zufällig entschieden und nach den ersten Tests mitgeteilt.

2. Welche Methoden werden verwendet und wie wird die Studie ablaufen?

Wenn sich aus den Online-Tests oder einer Untersuchung durch eine betreuende ÄrztIn der begründete Verdacht ergibt, dass es sich um Konzentrationsprobleme wegen Long COVID handeln könnte, werden Sie über die Studie aufgeklärt und um Zustimmung zur Teilnahme gebeten.

Das Studienteam wird Ihnen diese Aufklärungsunterlagen geben und in einem live-online Gespräch alle Studienmaßnahmen erklären.

Wenn Sie mit der Teilnahme an der Studie einverstanden sind, bekommen Sie eine Studien-Nummer. Alle Daten für die Studie werden dann nur mehr unter dieser Studiennummer gespeichert (pseudonymisiert).

Wenn zur Teilnahme zugestimmt wird, vereinbaren wir einen Online-Termin zu Testung von Ihrer Konzentrations- und Merkfähigkeit, Lebensqualität und Teilhabe im Alltag. Dadurch können wir einschätzen, wie schlimm die Einschränkungen durch Long COVID im Alltag sind und haben einen Ausgangswert, um abschätzen zu können, ob sich durch die online Ergotherapie etwas verbessert. Für die Tests und die spätere Ergotherapie brauchen Sie ein online-fähiges Gerät wie einen PC, oder ein Tablet mit Mikrofon und Kamera. Die Tests und auch die spätere Therapie werden ausschließlich von geschulten, zur Verschwiegenheit verpflichteten MitarbeiterInnen in einem geschützten digitalen Raum durchgeführt. Die Tests beim ersten Termin dauern etwa 30 Minuten.

Anschließend findet eine zufällige Zuordnung in die verschiedenen Studienarme (Randomisierung) statt. Zufällig wird entschieden, ob Sie in eine Interventions- oder in eine Kontrollgruppe kommen.

Während die Kontrollgruppe (50% aller TeilnehmerInnen) keine Behandlung erhält und nach zwölf und 24 Wochen erneut auf die Konzentrationsfähigkeit und Lebensqualität hin getestet wird (wieder mittels online-Termin, Dauer ca. 35 Minuten), erhalten die Interventionsgruppen (50% der Teilnehmenden) von Woche null bis zwölf jeweils zweimal pro Woche halbstündige, digitale Ergotherapie-Sessions. Dabei erhalten 25% aller Teilnehmenden live online Ergotherapie, treffen sich also online mit ErgotherapeutInnen (Praxis Timm-Cook, Hannover) und werden in Techniken geschult, die ihnen den Umgang mit den Konzentrationsproblemen im Alltag erleichtern.

Die andere Hälfte der Teilnehmenden in der Interventionsgruppe (25% Aller, die an der ersten Testung teilgenommen haben), erhalten digitale Ergotherapie mittels zuvor aufgenommenen, speziell auf Long COVID abgestimmten Ergotherapievideos (ebenfalls zweimal eine halbe Stunde pro Woche. Die Videos können „on-demand“ abgerufen werden.

Beide Interventionsgruppen erhalten auch Anleitungen zum regelmäßigen Training im Alltag, die helfen sollen, besser mit den Einschränkungen durch Long COVID zurecht zu kommen.

Zusätzlich zur Testung der kognitiven Fähigkeiten und Lebensqualität werden wir Sie bitten, uns Ihre Sicht zur Diagnostik, Behandlung sowie zur körperlichen und psychischen Gesundheit mitzuteilen. Wenn Sie einverstanden sind, werden wir dazu Fragebögen zur Beantwortung in pseudonymisierter Form aushändigen (online oder wenn gewünscht als Papierfragebogen).

Alle Daten werden pseudonymisiert, also ohne direkten Rückschluss auf die teilnehmende Person ausgewertet. Durch die Auswertung der Daten wollen wir erreichen, dass Long COVID bei Jugendlichen und Erwachsenen in Zukunft besser behandelt werden kann.

3. Welche Risiken entstehen bei einer Teilnahme für mich?

Risiken könnten sich theoretisch aus dem Datenschutz ergeben. Das wird in Teil 2 dieser Aufklärung genau erklärt.

4. Wann darf ich nicht an der Studie teilnehmen?

Von der Studienteilnahme ausgeschlossen sind:

- Alter unter 16 Jahre, zwischen 19-29 Jahre und über 51 Jahre,
- diagnostizierte kognitive Einschränkungen *vor* Infektion mit SARS-CoV2,
- die vorangegangene SARS-CoV2 wurde nicht mittels PCR- Test bestätigt,
- kein Zugang zu PC, Tablet, Smartphone mit Internetzugang,
- Kein Einverständnis zur Teilnahme.

5. Kann ich die Studie vorzeitig beenden?

Die Teilnahme an der Studie kann jederzeit ohne Angabe von Gründen beendet werden. Die Beendigung der Teilnahme hat keinerlei negative Effekte für Sie.

6. Welcher persönliche Nutzen entsteht für mich und bekomme ich eine Aufwandsentschädigung?

Durch die Teilnahme haben Sie die Chance, an einem neuartigen Therapieversuch für Long COVID teilzunehmen. Wir nehmen Long COVID ernst und hoffen, dass die Symptome durch eine strukturierte Online-Intervention gebessert werden können. Aber es muss allen Teilnehmenden klar sein, dass die Studie eine Pilot-Studie mit experimentellem Charakter ist. Das heißt, dass wir nicht sagen können, ob sich eine Besserung der Symptome von Long COVID durch die digitale Intervention ergibt. Wir können auch nicht sagen, inwiefern die Therapieart von den PatientInnen angenommen wird. Für den einzelnen Studienteilnehmer könnte die Teilnahme also möglicherweise von Vorteil sein, das übergeordnete Ziel der Studie ist es aber, Therapiemöglichkeiten bei Long COVID im Allgemeinen besser zu verstehen und die Versorgung für alle Jugendlichen (junge Erwachsene) und Erwachsenen mit dieser Erkrankung zu verbessern. Ob davon bereits Studienteilnehmer selbst profitieren, kann nicht vorhergesagt werden. Eine Aufwandsentschädigung ist nicht vorgesehen.

7. Was passiert, wenn sich durch die Untersuchung Zufallsbefunde ergeben?

Da es im Rahmen der Studie zu Ergebnissen bzw. Erkenntnissen kommen kann, die auf eine andere Erkrankung hindeuten, welche nicht Teil der Studie ist, bitten wir Sie uns vor Beginn der Studie mitzuteilen, ob Sie auf diese Befunde aufmerksam gemacht werden wollen (siehe Einwilligungserklärung). Je nach Befund ergeben sich daraus weitere Empfehlungen hinsichtlich Therapie oder weiterer Untersuchungen.

8. Gibt es eine Studien-/Unfall-/Wegeversicherung?

Da die Begleitstudie nur online stattfindet, wurde keine Studien-/ Unfall- oder Wegeversicherung abgeschlossen.

Teil II: Datenschutzerklärung

II. 1. Was geschieht mit meinen Daten?

Zum Zweck der Durchführung der klinischen Studie werden medizinische Befunde und persönliche Informationen (wie Alter, Geschlecht, Zeitpunkt der COVID-19 Erkrankung) über Sie erhoben und durch StudienmitarbeiterInnen in der Studiendatenbank dokumentiert.

Die für die klinische Studie wichtigen Daten werden nur in verschlüsselter (pseudonymisierter) Form in einer passwortgeschützten, elektronischen Datenbank gespeichert. Pseudonymisiert bedeutet, dass keine Angaben von Namen bzw. Initialen verwendet werden, sondern nur ein Zahlen- oder Buchstabencode. Die Daten sind gegen unbefugten Zugriff gesichert. Nur die Studienleitung vor Ort und deren Team werden in der Lage sein, Sie persönlich anhand der verschlüsselten Daten zu identifizieren. Die Weitergabe der Studiendaten an die Studienzentrale erfolgt in pseudonymisierter Form. Die Weitergabe der Studiendaten an Dritte erfolgt ausschließlich in anonymisierter Form; das bedeutet, dass eine Zuordnung zu Ihnen nicht mehr möglich ist.

Ihr Name und das Geburtsdatum werden auf der Einwilligungserklärung eingetragen, die getrennt von allen anderen Studiendaten aufbewahrt werden. Es ist möglich, dass InspektorInnen von offiziellen Überwachungsbehörden Einsicht in diese Dokumente nehmen, um die vorschriftsgemäße Durchführung der Studie zu überprüfen. InspektorInnen sind verpflichtet, Ihre persönlichen Daten vertraulich zu behandeln.

Die Rechtsgrundlage zur Verarbeitung der betreffenden personenbezogenen Daten ist Ihre freiwillige schriftliche Einwilligung gemäß DSGVO (nach Art. 6 Abs. 1 lit. a) DSGVO i. V. m. Art. 9 Abs. 2 lit. a) DSGVO) bei der Verarbeitung sensibler Daten. Ihre Einwilligung ist freiwillig und kann jederzeit ohne nachteilige Auswirkungen mit der Wirkung für die Zukunft widerrufen werden. Ohne Ihre Einwilligung zur Verarbeitung und Weitergabe der betreffenden Daten in verschlüsselter Form, können Sie/ kannst Du/ kann Ihr Kind nicht an der oben genannten klinischen Studie teilnehmen. Veröffentlichungen in Fachjournalen und öffentlichen Studienregistern (z. B. clinicaltrials.gov oder EU Clinical Trials Register) oder Präsentationen von Studienergebnissen werden keinerlei Daten beinhalten, anhand derer Sie persönlich identifiziert werden kann.

Ihre Daten werden in dieser Studie primär zum oben genannten Studienzweck, nämlich der Verbesserung von Diagnose und Therapie bei Kindern und Jugendlichen mit Long COVID, verarbeitet. Jedoch kann es vorkommen, dass im Laufe der Untersuchung und Datenauswertung weitere

Forschungsfragen aufkommen, die mit dem Gegenstand der hier vorliegenden Studie verwandt sind. In diesem Fall würden Deine Daten/die Daten Ihres Kindes ebenfalls für diesen Zweck verwendet werde. Sie können dem jedoch in der Einwilligung explizit widersprechen.

Ihre erhobenen Daten werden von der Studienzentrale bis 31.12.2023 gespeichert. Danach werden die Daten komplett anonymisiert bzw. gelöscht. Nach Anonymisierung oder Löschung ist ein Rückschluss auf Sie nicht mehr möglich. Die Verantwortung für die Datenverarbeitung im Rahmen dieser klinischen Studie liegt beim:

Prof. Georg Behrens, Klinik für Rheumatologie und Immunologie, Carl-Neuberg-Str. 1, 30625 Hannover

II. 3. Ergänzende Information gemäß Europäischer Datenschutz-Grundverordnung

Bezüglich der Daten haben Sie folgende Rechte, die Sie gegenüber dem Verantwortlichen geltend machen können:

Recht auf Auskunft: Sie haben das Recht auf Auskunft über die Sie betreffenden personenbezogenen Daten, die im Rahmen der klinischen Studie erhoben, verarbeitet oder ggf. an Dritte übermittelt werden (einschließlich einer kostenfreien Kopie). Auch können Sie die Überlassung eines tragbaren elektronischen Datenträgers, auf dem die Sie betreffenden Daten strukturiert und in einem gängigen Format (Office- oder PDF-Datei) gespeichert werden, oder die Übermittlung dieser Daten an einen anderen Verantwortlichen* verlangen (Artikel 15 DSGVO).

Recht auf Löschung: Sie haben das Recht auf Löschung Ihrer personenbezogener Daten, z. B. wenn diese Daten für den Zweck, für den sie erhoben wurden, nicht länger benötigt werden (Artikel 17 DSGVO).

Recht auf Einschränkung der Verarbeitung: Unter bestimmten Voraussetzungen haben Sie das Recht, eine Einschränkung der Verarbeitung zu verlangen, d. h. die Daten dürfen nur gespeichert, aber nicht verarbeitet werden. Dies müssen Sie beantragen (Artikel 18 DSGVO).

Recht auf Datenübertragbarkeit: Sie haben das Recht, die Sie betreffenden personenbezogenen Daten, die Sie dem* Verantwortlichen* für die klinische Studie bereitgestellt haben, zu erhalten. Damit können Sie beantragen, dass diese Daten (strukturiert und in einem gängigen Format auf einem tragbaren elektronischen Datenträger) entweder Ihnen oder einem anderen von Ihnen benannten (weiteren) Verantwortlichen für die Datenverarbeitung im Sinne der DSGVO übermittelt werden können (Artikel 20 DSGVO).

Widerspruchsrecht: Sie haben das Recht, jederzeit gegen konkrete Entscheidungen oder Maßnahmen zur Verarbeitung der Sie betreffenden personenbezogenen Daten Widerspruch einzulegen. Eine Verarbeitung (neuer Daten) findet anschließend nicht mehr statt, es sei denn, die Verarbeitung ist gesetzlich weiterhin gefordert – wie im Arzneimittelgesetz AMG (Artikel 21 DSGVO). Möchten Sie diese Rechte in Anspruch nehmen, wenden Sie sich bitte an Ihren* Prüfer* oder an den* Datenschutzbeauftragten* Ihres Prüfzentrums.

Einschränkungen: Wir möchten Sie an dieser Stelle darauf hinweisen, dass die aufgeführten Rechte eingeschränkt werden können, wenn diese Rechte die Verwirklichung der Forschungszwecke unmöglich machen oder ernsthaft beinträchtigen und die Beschränkung für die Erfüllung der Forschungszwecke notwendig ist (Artikel 89 DSGVO, §27 BDSG-neu). Die Rechte Ihres Kindes auf Auskunft, Datenübertragbarkeit und Berichtigung fehlerhaft verarbeiteter Daten bestehen nicht, sofern die Auskunftserteilung einen unverhältnismäßigen Aufwand erfordern würde oder technisch unmöglich ist. Ob die Rechte Ihres Kindes eingeschränkt werden können, bedarf einer konkreten Abwägung.

Sie haben das **Recht, Beschwerde bei einer Aufsichtsbehörde einzulegen,** wenn Sie der Ansicht sind, dass die Verarbeitung der Sie betreffenden personenbezogenen Daten gegen die DSGVO verstößt.

Datenschutzbeauftragter* / Datenschutz-Aufsichtsbehörde

Landesbeauftragte für den Datenschutz Niedersachsen (LfD)

Postfach 221

30002 Hannover

Tel.: 0511 120-4500, Fax: 0511 120-4599

E-Mail: poststelle@lfd.niedersachsen.de

Datenschutzbeauftragte/r der Medizinischen Hochschule Hannover

Carl-Neuberg-Str. 1

30625 Hannover

Tel.: 0511- 532-2555

E-Mail: Datenschutz@mh-hannover.de

____

(Datum, Name & Unterschrift Versuchsleiter*in bzw. Prüfarzt*in)

____

(Datum, Name & Unterschrift Teilnehmer*in)

Anhang 3: Einverständniserklärung Jugendliche ab 16. Lebensjahr (inkl. Elterneinverständniserklärung)

Studienleitung
PD Dr. Alexandra Dopfer-Jablonka

Zentrale Kontaktstelle
PD Dr. med. Alexandra Dopfer-Jablonka
Klinik für Immunologie und Rheumatologie - OE 6830
Medizinische Hochschule Hannover
Carl Neuberg Straße 1
30625 Hannover
Email: Jablonka.Alexandra@mh-hannover.de
Tel: +49 511 532 3014

Einwilligung für Jugendliche ab 16 Jahren zur Studie: „ErgoLoCo" – online Ergotherapie bei Long COVID

TEIL A: Einverständnis Jugendliche

Teil B: Einverständnis Sorgeberechtigte

TEIL A: durch die/den Jugendlichen zu unterzeichnen

Ich habe die Aufklärung über die Studie inklusive der Datenschutzerklärung gelesen und zur Kenntnis genommen. Meine eventuellen Rückfragen konnten mir durch die für die Untersuchung zuständige Person zufriedenstellend beantwortet werden und ich hatte ausreichend Zeit, meine Teilnahme an dem Vorhaben zu überdenken.

....	....	I_I_I I_I_I I_I_I_I_I
Vorname des Teilnehmenden	Name des Teilnehmenden	Geburtsdatum

Im Folgenden gebe ich mein Einverständnis für die angekreuzten Punkte:

- □ Teilnahme an der Studie mit dem Wissen, dass die Teilnahme an der Studie jederzeit von mir beendet werden kann.
- □ Verarbeitung meiner Daten nur zu Studienzwecken und Zwecken, die mit dem Studienziel verwandt sind.
- □ Einladung zur Beantwortung von Fragebögen und zur Kontaktaufnahme, um ggf. weitere Studientermine zu vereinbaren.

Falls sich im Laufe der Untersuchung oder bei der Auswertung meiner Daten medizinisch relevante Zufallsbefunde ergeben, möchte ich, dass Folgendes geschieht:

- □ Zufallsbefunde sollen mir mitgeteilt werden.
- □ Zufallsbefunde sollen mir <u>nicht</u> mitgeteilt werden.

Im Falle eines Widerrufs meiner Einwilligung zur Studienteilnahme:

- □ Dürfen alle meine bisher erhobenen Daten für die Zwecke dieser Studie und Studienzwecken und Zwecken, die mit dem Studienziel verwandt sind, weiterverwendet werden.
- □ Dürfen meine bisher erhobenen Daten für die Zwecke dieser Studie weiterverwendet werden.
- □ Müssen alle meine nicht mehr benötigten Daten unverzüglich gelöscht werden.

Meine Einwilligung zur Studienteilnahme ist freiwillig und ich kann diese jederzeit ohne Angabe von Gründen für die Zukunft widerrufen. Durch den Widerruf der Einwilligung wird die Rechtmäßigkeit der aufgrund der Einwilligung bis zum Widerruf erfolgten Verarbeitung nicht berührt.

____

(Datum, Name & Unterschrift *Prüfarzt*in*)

____

(Datum, Name & Unterschrift des 17-18-jährigen Teilnehmenden)

Name, Vorname: ____

Studien-ID des Teilnehmers:
vom Arzt*in* auszufüllen

-- -- -- --	-- -- -- --	-- -- -- --	-- -- -- --

TEIL B: durch die/den Sorgeberechtigten zu unterzeichnen

Ich habe die Aufklärung über die Studie inklusive der Datenschutzerklärung gelesen und zur Kenntnis genommen. Meine eventuellen Rückfragen konnten mir durch die für die Untersuchung zuständige Person zufriedenstellend beantwortet werden und ich hatte ausreichend Zeit, die Teilnahme meines Kindes an dem Vorhaben zu überdenken.

....	....	I_I_I I_I_I I_I_I_I_I
Vorname des Teilnehmenden	Name des Teilnehmenden	Geburtsdatum

Im Folgenden gebe ich mein Einverständnis für die angekreuzten Punkte:

- □ Teilnahme meines Kindes an der Studie mit dem Wissen, dass die Teilnahme an der Studie jederzeit von mir beendet werden kann.

- ☐ Verarbeitung der Daten, die während der Teilnahme meines Kindes erhoben werden nur zu Studienzwecken und Zwecken, die mit dem Studienziel verwandt sind.

- ☐ Einladung meines Kindes zur Beantwortung von Fragebögen und zur Kontaktaufnahme, um ggf. weitere Studientermine zu vereinbaren.

Falls sich im Laufe der Untersuchung oder bei der Auswertung der Daten meines Kindes medizinisch relevante Zufallsbefunde ergeben, möchte ich, dass Folgendes geschieht:

- ☐ Zufallsbefunde sollen mir oder meinem Kind mitgeteilt werden.
- ☐ Zufallsbefunde sollen mir oder meinem Kind nicht mitgeteilt werden.

Im Falle eines Widerrufs meiner Einwilligung oder dem Widerruf der Einwilligung durch mein Kind zur Studienteilnahme:

- ☐ Dürfen alle bisher erhobenen Daten meines Kindes für die Zwecke dieser Studie und Studienzwecken und Zwecken, die mit dem Studienziel verwandt sind weiterverwendet werden.
- ☐ Dürfen meine bisher erhobenen Daten meines Kindes für die Zwecke dieser Studie weiterverwendet werden.

- ☐ Müssen alle nicht mehr benötigten Daten meines Kindes unverzüglich gelöscht werden.

Die Einwilligung zur Studienteilnahme ist freiwillig und ich oder mein Kind können diese jederzeit ohne Angabe von Gründen für die Zukunft widerrufen. Durch den Widerruf der Einwilligung wird die Rechtmäßigkeit der aufgrund der Einwilligung bis zum Widerruf erfolgten Verarbeitung nicht berührt.

____

(Datum, Name & Unterschrift *Prüfarzt*in*)

____

(Datum, Name & Unterschrift des Sorgeberechtigten)

Name, Vorname: ____

Studien-ID des Teilnehmers:
vom Arzt**in* auszufüllen

-- -- -- --	-- -- -- --	-- -- -- --	-- -- -- --

Anhang 4: Einverständniserklärung Erwachsene

Studienleitung
PD Dr. Alexandra Jablonka

Zentrale Kontaktstelle
PD Dr. med. Alexandra Jablonka
Klinik für Immunologie und Rheumatologie - OE 6830
Medizinische Hochschule Hannover
Carl Neuberg Straße 1
30625 Hannover
Email: Jablonka.Alexandra@mh-hannover.de
Tel: +49 511 532 3014

Einwilligung für Erwachsene zur Studie: „ErgoLoCo“ – online Ergotherapie bei Long COVID

Ich habe die Aufklärung über die Studie inklusive der Datenschutzerklärung gelesen und zur Kenntnis genommen. Meine eventuellen Rückfragen konnten mir durch die für die Untersuchung zuständige Person zufriedenstellend beantwortet werden und ich hatte ausreichend Zeit, meine Teilnahme an dem Vorhaben zu überdenken.

....	....	I_I_I I_I_I I_I_I_I_I
Vorname des Teilnehmenden	Name des Teilnehmenden	Geburtsdatum

Im Folgenden gebe ich mein Einverständnis für die angekreuzten Punkte:

- ☐ Teilnahme an der Studie mit dem Wissen, dass die Teilnahme an der Studie jederzeit von mir beendet werden kann.
- ☐ Verarbeitung meiner Daten nur zu Studienzwecken und Zwecken, die mit dem Studienziel verwandt sind.
- ☐ Einladung zur Beantwortung von Fragebögen und zur Kontaktaufnahme, um ggf. weitere Studientermine zu vereinbaren.

Falls sich im Laufe der Untersuchung oder bei der Auswertung meiner Daten medizinisch relevante Zufallsbefunde ergeben, möchte ich, dass Folgendes geschieht:

- ☐ Zufallsbefunde sollen mir mitgeteilt werden.
- ☐ Zufallsbefunde sollen mir nicht mitgeteilt werden.

Im Falle eines Widerrufs meiner Einwilligung zur Studienteilnahme:

- ☐ Dürfen alle meine bisher erhobenen Daten für die Zwecke dieser Studie und Studienzwecken und Zwecken, die mit dem Studienziel verwandt sind weiterverwendet werden.
- ☐ Dürfen meine bisher erhobenen Daten für die Zwecke dieser Studie weiterverwendet werden.

- ☐ Müssen alle meine nicht mehr benötigten Daten unverzüglich gelöscht werden.

Meine Einwilligung zur Studienteilnahme ist freiwillig und ich kann diese jederzeit ohne Angabe von Gründen für die Zukunft widerrufen. Durch den Widerruf der Einwilligung wird die Rechtmäßigkeit der aufgrund der Einwilligung bis zum Widerruf erfolgten Verarbeitung nicht berührt.

____

(Datum, Name & Unterschrift *Prüfarzt*in*)

____

(Datum, Name & Unterschrift des Teilnehmenden)

Name, Vorname: ____

Studien-ID des Teilnehmers:
vom Arzt**in* auszufüllen

-- -- -- --	-- -- -- --	-- -- -- --	-- -- -- --

Anhang 5: Angaben zum Testinstrument COPM

Die COPM (Canadian Occupational Performance Measure) Testung erfasst strukturiert die Selbsteinschätzung der Betätigungsperformanz und Zufriedenheit. Die Testung wird durch die ProbandInnen online unter Anleitung durch eine qualifizierte StudienmitarbeiterIn durchgeführt. Die Dauer beträgt ca. 30 Minuten. Hier eine Übersicht über die erhobenen Daten:

Anhang 6: Angaben zum Testinstrument WIT-22

Der Wilde Intelligenztest 2 ist ein Instrument zur Erfassung der allgemeinen Intelligenz mit besonderem Bezug zu beruflicher Arbeit. Der WIT-2 besteht aus 11 Modulen zu unterschiedlichen Facetten von Intelligenz und Arbeitsverhalten. In der Studie werden davon zwei Module genutzt. Das Modul MF erfasst die Merkfähigkeit und besteht aus einer Einpräge- und einer Abrufphase. Zwischen diesen beiden Phasen wird als Distraktor das Modul EM zur Erfassung der kognitiven Arbeitseffizienz eingesetzt. Die Testung erfolgt im Hogrefe-Testsystem und wird online unter Anleitung furch eine qualifizierte StudienleiterIn durchgeführt. Die Testdauer beträgt einschließlich der Instruktionen ca 35 min.

Anhang 7: Angaben zum Testinstrument IMET

Der IMET (Index zur Messung von Einschränkungen der Teilhabe) erhebt die Einschränkungen der Teilhabe im Alltag und wird über das Corona-Defeat Portal automatisch erhoben. Die Testung wird durch die ProbandInnen online selbstverantwortlich in einem vorgefertigten Klick-Fragebogen durchgeführt. Die Dauer beträgt ca. 10 Minuten.

Anhang 8: Angaben zum Testinstrument NeuroQual

Der NeuroQual erhebt Daten zur kognitiven Leistungsfähigkeit und Fatigue und diesbezüglichen Lebensqualität. Der NeuroQaul wird in deutscher Sprache zur Verfügung gestellt. Die Testung wird durch die ProbandInnen online durchgeführt. Die Dauer beträgt ca. 5-10 Minuten

Anhang 9: Fragen zur Prozessevaluation

Wie konnten Sie den Inhalten des letzten Moduls (letzten vier Therapieeinheiten) folgen?

☐ überhaupt nicht ☐ eher nicht ☐ etwas ☐ eher gut ☐ gut

Konnten Sie die Inhalte der des letzten Moduls in ihrem Alltag anwenden?

☐ überhaupt nicht ☐ eher nicht ☐ etwas ☐ eher gut ☐ gut

Hat sich Ihr Handlungsproblem innerhalb des letzten Moduls verändert?

☐ verschlechtert ☐ eher verschlechtert ☐ gleich geblieben ☐ eher verbessert ☐ verbessert

Was war im letzten Modul besonders hilfreich für Sie?

Freitextfeld

Sind während des letzten Moduls Probleme (jeglicher Art) aufgetreten?

Freitextfeld

Wie zufrieden waren Sie mit dem letzten Modul?

☐ überhaupt nicht zufrieden ☐ eher nicht zufrieden ☐ neutral ☐ eher zufrieden ☐ zufrieden

Zentrum für Innere Medizin
Klinik für Rheumatologie und Immunologie

MHH, Zentrum für Innere Medizin, OE 6830, 30625 Hannover

OE 6830
PD Dr. med Alexandra Jablonka
Tel.: 0511 532- 5337
0170 - 3805936
Fax: 0511 532-8055
jablonka.alexandra@mh-hannover.de

Carl-Neuberg-Straße 1
30625 Hannover
Telefon: 0511 532-0
www.mh-hannover.de

An die
Ethikkommission der MHH
Prof. Dr. med. Bernhard Schmidt
OE 9515

9. Mai 2024

–

Amendement zum Ethikantrag DEFEnse Against COVID-19 Study – Looking forward: DEFEAT Corona 9948_BO_K_2021; Ergänzung im Rahmen des Teilprojekts ErgoLoCo

Sehr geehrter Herr Professor Schmidt,
sehr geehrte Mitglieder der Ethikkommission,

zu unserer Studie DEFEnse Against COVID-19 STudy – Looking forward möchten wir gerne eine weitere Ergänzung vornehmen, für deren Genehmigung wir sehr dankbar wären:

Im Rahmen unserer Studie und der Rekrutierung haben wir bemerkt, dass die Rekrutierung von Kindern und Jugendlichen sehr schwierig ist. Wir haben bereits umfangreiche, deutschlandweite Werbung via Social Media, Schulen, Kinderärzt:innen und Long COVID Ambulanzen durchgeführt. Da die von Long COVID betroffenen Kinder und Jugendlichen im Gegensatz zu unserer ersten Annahme vermehrt an Fatigue leiden, aber nur relativ geringe Einschränkungen im Bereich Kognition und Merkfähigkeit berichten, gibt es nur sehr wenige Jugendliche, deren Beschwerden auf unser Rekrutierungsschema zutreffen.

Außerdem sind die Kinder und Jugendlichen in Bezug auf Ergotherapie exzellent versorgt, ca. 90% haben bereits eine Ergotherapeutische Interventionen hinter sich oder diese sind geplant. Außerdem haben wir gerade in der Kontrollgruppe eine hohe Dropout-Rate, da die Kinder dann doch Ergotherapie außerhalb der Studie erhalten und nicht die vorgesehene Wartezeit verstreichen lassen wollen. Dies ist natürlich wünschenswert in Hinblick auf die Versorgung dieser Patientengruppe, aber gefährdet unser Studienziel.

Auf Grund der Kürze des Rekrutierungszeitraumes würden wir darum bitten das Studiendesign leicht zu verändern und möchten in die Gruppe der Kinder und Jugendlichen auch junge Erwachsene einzubeziehen, welche nach Einschätzung der Ergotherapeut:innen auch im Hinblick auf Ihre Erfahrungswelt den Jugendlichen sehr ähnlich sind. Wir möchten Jugendliche und junge Erwachsene im Alter zwischen16 und 25 Jahren (zuvor 16-18 Jahre) rekrutieren. Die sonstigen Studienzwecke und das Design ändern sich nicht. Auch müssen die Teilnehmer:Inneninformationen oder Einverständniserklärungen nicht angepasst werden, da die bisherigen Dokumente alle Altersstufen ab 16 Jahren abdecken. Die Anzahl in den Gruppen und bislang genehmigten Proband:Innen pro Gruppe würde durch diese Änderung ebenfalls nicht berührt.

Zusammenfassend möchten wir beantragen, die zwei Rekrutierungsgruppen von bisher Jugendlichen (16-18 Jahre) und Erwachsene (30-50 Jahre) zu ändern in: Jugendliche und junge Erwachsene (16-25 Jahre) und Erwachsene (30-50 Jahre).

Ihre Zustimmung würde uns ermöglichen das Projekt zeitgerecht abzuschließen und die Aussagekraft und Power der Studie zu erhalten. Außerdem spiegelt dies den Versorgungsbedarf bei Long COVID besser wieder. Für Rückfragen stehen wir stets gern zur Verfügung.

Mit freundlichen Grüßen

Alexandra Dopfer-Jablonka

Zentrum für Innere Medizin
Klinik für Rheumatologie und Immunologie

MHH, Zentrum für Innere Medizin, OE 6830, 30625 Hannover

OE 6830
PD Dr. med Alexandra Jablonka
Tel.: 0511 532- 5337
0170 - 3805936
Fax: 0511 532-8055
jablonka.alexandra@mh-hannover.de

Carl-Neuberg-Straße 1
30625 Hannover
Telefon: 0511 532-0
www.mh-hannover.de

An die
Ethikkommission der MHH
Prof. Dr. med. Bernhard Schmidt
OE 9515

9. Mai 2024

–

Amendement zum Ethikantrag DEFEnse Against COVID-19 Study – Looking forward: DEFEAT Corona 9948_BO_K_2021; Ergänzung im Rahmen des Teilprojekts ErgoLoCo

Sehr geehrter Herr Professor Schmidt,
sehr geehrte Mitglieder der Ethikkommission,

zu unserer Studie DEFEnse Against COVID-19 STudy – Looking forward möchten wir gerne eine weitere Ergänzung vornehmen, für deren Genehmigung wir sehr dankbar wären:

Im Rahmen unserer Studie und der Rekrutierung haben wir bemerkt, dass bei einem hohen Anteil der ProbandInnen, die aufgrund von Long-COVID bei uns Hilfe suchen, im Rahmen der SARS-CoV-2 Infektion kein PCR-Nachweis erfolgte. Nach langen Jahren der Pandemie wurden viele Infektionen ausschließlich mittels Antigen-Schnelltest oder Antikörpernachweis nachgewiesen.

Gerne möchten wir daher unsere **Rekrutierungsstrategie anpassen** und das Einschlusskriterium „nachgewiesene Coronainfektion" auf **den Nachweis einer SARS-CoV-2 Infektion mittels PCR, Antigenschnelltest oder Antikörpertest** ausweiten. Die Anpassung spiegelt die Testrealität bei Long COVID Patient*Innen besser wieder. Das Studienziel, die Gruppengrößen oder andere Faktoren in unserer Studie ändern sich dadurch ausdrücklich nicht.

Ihre Zustimmung würde uns ermöglichen das Projekt noch besser auf die Bedürfnisse von Betroffenen abzustimmen, es zeitgerecht abzuschließen und die Aussagekraft und Power der Studie zu erhalten. Für Rückfragen stehen wir stets gern zur Verfügung.

Mit freundlichen Grüßen

Alexandra Dopfer-Jablonka

Studienleitung

Zentrum für Innere Medizin
Klinik für Rheumatologie und Immunologie

MHH, Zentrum für Innere Medizin, OE 6830, 30625 Hannover

OE 6830
PD Dr. med Alexandra Jablonka
Tel.: 0511 532- 5337
0170 - 3805936
Fax: 0511 532-8055
jablonka.alexandra@mh-hannover.de

Carl-Neuberg-Straße 1
30625 Hannover
Telefon: 0511 532-0
www.mh-hannover.de

An die
Ethikkommission der MHH
Prof. Dr. med. Bernhard Schmidt
OE 9515

9. Mai 2024

–

Amendement zum Ethikantrag DEFEnse Against COVID-19 Study – Looking forward: DEFEAT Corona 9948_BO_K_2021; Ergänzung im Rahmen des Teilprojekts ErgoLoCo

Sehr geehrter Herr Professor Schmidt,
sehr geehrte Mitglieder der Ethikkommission,

zu unserer Studie DEFEnse Against COVID-19 STudy – Looking forward möchten wir gerne eine weitere Ergänzung vornehmen, für deren Genehmigung wir sehr dankbar wären:

Im Rahmen unserer Studie und der Rekrutierung haben wir bemerkt, dass ein hoher Anteil der ProbandInnen, die aufgrund von Long-COVID bei uns Hilfe suchen, zwischen 30 und 40 Jahren alt sind. Der Altersmedian der PatientIn-nen, die an unserer Studie teilnehmen wollen, liegt bei etwa 35 Jahren.

Gerne möchten wir daher unsere **Rekrutierungs- und Auswertungsstrategie anpassen** und die Gruppenverteilung insofern ändern, als dass wir **n=80 ProbandInnen zwischen 16 und 35 Jahren** (zuvor n=80 ProbandInnen zwischen 16 und 30 Jahren) und wir **n=80 ProbandInnen zwischen 35 und 70 Jahren** (zuvor n=80 ProbandInnen zwischen 30 und 50 Jahren) einschließen.

Die Anpassung spiegelt den Versorgungsbedarf bei Long COVID besser wieder. Ihre Zustimmung würde uns ermög-lichen das Projekt noch besser auf die Bedürfnisse von Betroffenen abzustimmen, es zeitgerecht abzuschließen und die Aussagekraft und Power der Studie zu erhalten. Für Rückfragen stehen wir stets gern zur Verfügung.

Mit freundlichen Grüßen

Alexandra Dopfer-Jablonka

Studienleitung
