# Supplementary material for: Improvement in quality of life and cognitive function in Post-COVID syndrome after online occupational therapy: Results from a randomized controlled pilot study
Source: PLoS One. 2025 May 20;20(5):e0312714. doi: 10.1371/journal.pone.0312714 (PMC12091760; doi:10.1371/journal.pone.0312714)
Supplement: S2 File — (PDF) [file pone.0312714.s003.pdf]

English translation of study protocol and supplementary material and amendments

“Improvement in quality of life and cognitive function in Post Covid Syndrome after online occupational therapy: results from a 3 randomized controlled pilot study “ by Schröder et al.

This document contains:

- Study protocol (pages 2-15)
- Supplementary material (pages 16-37)
- Amendments to the study protocol (pages 38-40)

Ethics Committee of Hannover Medical School
(for information to the Ethics Committee of the University Medical Center Göttingen)

English translation of the Study protocol

for the study
ErgoLoCo - development of a digital occupational therapy intervention for long COVID patients

Clinic for Rheumatology and Immunology
Hannover Medical School

together with
Institute for General Medicine Göttingen
and
Ostfalia University of Applied Sciences Braunschweig

Study director
PD Dr. med. Alexandra Jablonka
Clinic for Immunology and Rheumatology - OE 6830
Hanover Medical School
Carl Neuberg Street 1
30625 Hanover
Email: Jablonka.Alexandra@mh-hannover.de
Phone: +49 511 532 3014

Applicants

UMG:	MHH:	Ostfalia:
Dr. med. Frank Müller	PD Dr. med. Alexandra Dopfer-Jablonka	Prof. Dr. Frank Klawonn
Prof. Dr. Eva Hummers	Prof. Dr. med. Georg Behrens	
	Prof. Dr. med. Sandra Steffens	
	Prof. Dr. med. Christine Happle, PhD	

Hannover, Göttingen, Wolfenbüttel, June 2022

Content

1. Objective and justificaton of the study 4

1.1 State of research.......... 4

1.2 Justification for the study to be carried out: Benefit for medicine or scientific knowledge value 5

1.3 Objectives and scientific questions 5

2. Branches of study and overview of studies.......... 6

3. **Description of the intervention** 7

4.1 **Non-blinded randomized controlled pilot study** Characterization of the participants 7

4.2 Recruitment.......... 8

4.3 Information and consent.......... 8

4.4 Participation and revocation of participation 8

4.5 Study workflow - procedure and implementation.......... 8

4.6 Pseudonymization and data protection.......... 10

5. List of appendices.......... 12

6. Bibliography.......... 13

7. Appendices 15

Appendix 1: Information for adolescents from the age of 16 (including information for parents) 15

Appendix 2: Information for adults 21

Appendix 3: Declaration of consent for adolescents aged 16 and over (incl. parental consent form) 27

Appendix 4: Declaration of consent for adults 30

Appendix 5: Information on the COPM test instrument 32

Appendix 6: Information on the WIT-2 test instrument.......... 32

Appendix 7: Information on the IMET test instrument 33

Appendix 8: Information on the NeuroQuoL test instrument.......... 34

Appendix 9: Questions on process evaluation.......... 35

1. Objective and justification of the study

Long COVID is a new collective term developed during the current pandemic for long-term symptoms that persist or occur after COVID-19. The patho-mechanisms that lead to Long COVID are not yet clear, which means that therapeutic options are very limited. Long COVID manifests in symptom clusters that usually overlap and vary in intensity (Wong-Chew et al. 2022). The productive and social life of patients, as well as their subjective well-being, are often severely impaired by Long COVID over longer periods of time. Studies on the prevalence and clinical and socio-economic aspects of Long COVID show that Long COVID patients have severe impairments in their social and family life. One of the main symptoms in people who suffered from COVID-19 with persisting complaints is reduced cognitive performance, which mainly manifests as "brain fog", i.e. difficulty concentrating and fatigue. These symptoms can lead to problems in coping with daily activities and significantly impair recovery (Hugon et al. 2022).

There are currently no evidence-based treatment options for Long COVID with validated treatment benefit. The British National Institute for Health and Care Excellence (NICE) recommends integrating multidisciplinary treatment methods aimed at symptom management and reducing functional limitations (NICE 2022). Occupational therapy is considered an integral part of such multimodal, treatment concepts [AOTA: Occupational therapy in the age of Coronavirus] (Margetis et al. 2021; AOTA 2021; Belli et al. 2020). In Germany, occupational therapy can be prescribed by doctors for treatment of Long COVID over a period of twelve weeks (KBV, 2021). Studies support the effectiveness of occupational therapy in various clinical and rehabilitative settings for the treatment of daily limitations and symptoms similar to those of Long COVID patients (Kos et al. 2016).

On this basis and through the participatory involvement of stakeholders (affected persons, their relatives, medical professionals), a digital occupational therapy intervention for the treatment of everyday impairments in Long COVID was developed and will be tested and evaluated as part of the study project proposed here. The new form of treatment will be aimed at adolescents and adults. Occupational therapy is to be delivered as live online occupational therapy for one intervention group and via pre-recorded videos for another. Before and after the intervention, the test subjects will be tested on their cognitive abilities and questioned on their quality of life and behavior.

1.1 State of research

1.1.1 Long COVID in adults, children and adolescents

The term Long COVID describes symptoms that persist or appear more than four weeks after the onset of a SARS-CoV-2 infection (Koczulla et al. 2021). Symptoms are commonly referred to as post-COVID if their onset occurred in association with an acute SARS-CoV-2 infection, are still present more than twelve weeks later, last at least two months or occur recurrently and with varying severity and cannot be explained for any other reason (Nalbandian et al. 2021). A systematic review showed 55 different long-term effects of COVID-19 (Lopez-Leon et al. 2021). Common symptoms include shortness of breath, headaches, coughing, fatigue, and cognitive impairment such as brain fog. Persistent symptoms after COVID-19 can have a significant impact on the return to everyday life and work, which often also has financial consequences (Davis, et al., 2021). A recent study from the Netherlands shows that more than a third of all children and adolescents with Long COVID or Post COVID also show severe impairments in everyday life due to poor concentration (Brackel et al. 2021). A study from Great Britain also confirms that these symptoms often occur in adolescents (Miller et al. 2021). To date, there is no evidence-based treatment for these symptoms, neither for adolescents nor for adults.

1.1.2 Occupational therapy interventions for Long COVID

Current German and international guidelines in recommend holistic, person-centered approaches to the assessment and treatment of patients affected by long COVID. The NICE guidelines refer to the use of multidisciplinary rehabilitation approaches to promote symptom management and minimize functional limitations (NICE 2022). In particular, it should be taken into account that persistent symptoms affect everyday life and daily activities such as work, education, mobility, autonomy in daily activities, and psychological well-being. In this context, occupational therapy may be a helpful tool. Occupational therapy helps people of all ages to regain or compensate for important everyday roles and activities that are restricted due to functional impairments (DVE). This includes, among other benefits, restoration of the ability to act and the ability to perform existential and responsible tasks, such as returning to work, training, or school. Occupational therapy puts a particular focus on identifying new strategies for action and participation that enable patients to regain or maintain everyday activities in various areas of life (Le Granse et al.). New data shows that occupational therapy for long COVID is effective in overcoming everyday activity problems (Vij 2021).

1.2 Justification for the study to be carried out: Benefit for medicine or scientific knowledge value

The development of suitable therapies for the treatment of Long COVID is of acute and central importance, as the disease leads to long term restrictions in all areas of life in an increasing number of patients, with a particular impact on quality of life and productive activity (Townsend et al. 2020). Long COVID is a comparatively new disease, and it´s prevalence cannot yet be accurately estimated. However, it is estimated that around 7% of all SARS-CoV-2 infected people are at risk (Al-Aly et al. 2022). As a result, doctors around the world are confronted with a high number of patients with a plethora of additional patients expected in upcoming years.

The here proposed study is designed to evaluate a digital intervention for the treatment of Long COVID patients. The advance of digitalization brings new challenges and opportunities to the healthcare sector, including the development and provision of modern, resource-saving, and cost-effective healthcare services (Walzer 2022). Given the current pandemic, since 2020 teletherapy services have been increasingly used in the healthcare sector, with broad patient groups reached and successfully treated (Kataria und Ravindran 2018). However, it became clear that a targeted and evidence-based development of structured telemedicine approaches is necessary to ensure the quality of such treatment concepts (Peine et al. 2020).

Regarding treatment of Long COVID, it can be expected that digital treatment options represent a low-threshold and well-received offer as Long COVID patients can receive it in their immediate everyday environment. Factors such as lack of personnel capacity, regional medical undersupply and a shortage of specialists in the healthcare sector can be at least partially mitigated by digital mediation.

The evaluation of a digital occupational therapy intervention for Long COVID therefore seems sensible and important. If the intervention would be effective in the treatment of Long COVID, this would be helpful for many patients in Germany and beyond and may contribute significantly to the reduction of morbidity and care costs associated with COVID-19.

1.3 Objectives and scientific questions

The aim of this study is to evaluate a digital, occupational therapy intervention for the treatment of everyday impairments due to Long COVID-associated cognitive disorders such as brain fog, concentration disorders and fatigue. Specifically, the following hypotheses are to be tested,

1. Is an online occupational therapy intervention accepted by Long COVID patients?
2. Does an online occupational therapy intervention lead to a reduction in objectively measurable and subjectively perceived long COVID-associated activity restrictions in everyday life?
3. Does an online occupational therapy intervention lead to a reduction in objectively measurable and subjectively perceived cognitive impairments?
4. Which factors are effective in this digital occupational therapy intervention and which hinder its success?

2. Branches of study and overview of studies

The pilot study will be conducted as a randomized, controlled, unblinded interventional study.

It will be conducted in two equally sized groups: n=80 adults aged between 30 and 50 years (n=20 will receive live online occupational therapy, n=20 will receive occupational therapy via pre-recorded, on-demand videos, n=40 controls will not receive therapy) and n=80 adolescents aged between 16 and 18 years (n=20 will receive live online occupational therapy, n=20 will receive occupational therapy via pre-recorded, on-demand videos, n=40 controls will not receive therapy).

- ***FOR CHANGES OF THIS RECRUITMENT STRATEGY (Adaptation of age groups) PLEASE REFER TO THE AMENDEMENTS AT THE END OF THIS PROTOCOL -***

Recruitment of subjects, inclusion, testing and therapy should take place online via protected, digital channels. All n=160 subjects will receive cognitive testing at three time points: Start time at inclusion (t0), twelve weeks later (t1) and at week 24 (t2, end of study). Randomization into the different treatment groups takes place at t0 after the first cognitive test.

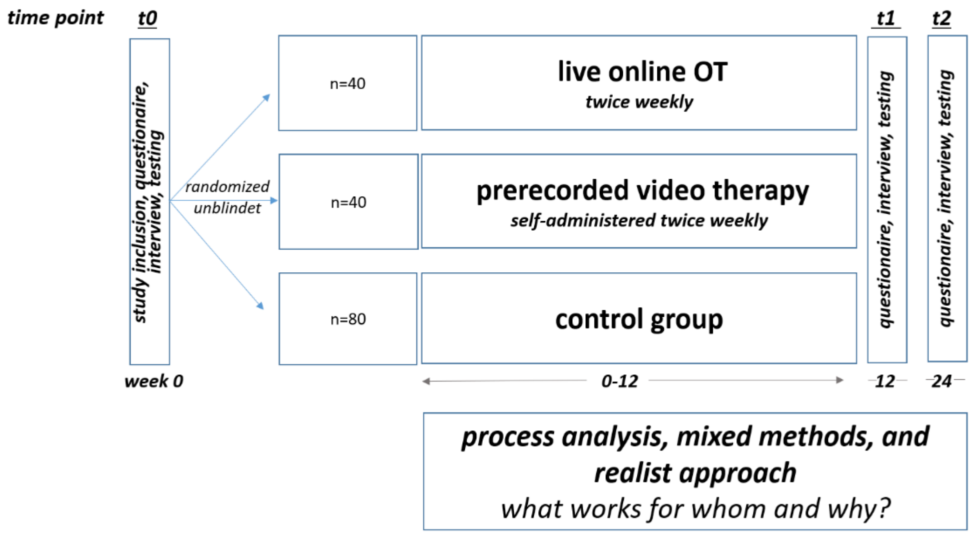

Abb. 1: *Overview of the planned intervention. n=160 subjects will be recruited online. At time (t) 0, they will receive a cognitive test and analysis of their abilities and complaints in everyday life and will then be randomized*

into the study arms. The intervention groups will each receive occupational therapy twice a week (live online or via pre-recorded videos). Twelve (t1) and 24 weeks (t2) later, all subjects will be tested again for their cognitive abilities and complaints in everyday life and asked about their experiences with the intervention.

3. Description of the intervention

A structured approach ensures that the intervention is designed to suit the target group, is based on the latest evidence and incorporates the experience of experts.

The intervention follows a modular structure with six consecutive therapy modules, each consisting of four 30-minute intervention units. Each module consists of a combination of therapy units led by occupational therapists and instructions for self-training. After completing the last module, participants in both interventional groups complete a full occupational therapy process including evaluation, analysis, adaptation, and re-evaluation of the occupational problem.

The intervention will be delivered via the online portal of the previously established digital coronavirus platform DEFEAT of Hannover Medical School (www. defeat-corona.de). For the intervention, the first interventional group will be offered two live online treatments by specially trained occupational therapists (occupational therapists from the Timm-Cook Hannover practice) over a period of twelve weeks. The second intervention group receives access to two weekly, non-individualized, on-demand video occupational therapy modules (recorded with simulated patients and therapists from the Timm-Cook Hannover practice) over a period of twelve weeks. The content of live online occupational and video modules have an identical structure and follow a therapy manual developed specifically for this study for digital application.

The occupational therapy approach is designed to treat Long COVID-associated cognitive impairments such as brain fog, fatigue, and/or problems with everyday activities caused by concentration disorders.

4.1 **Non-blinded randomized controlled pilot study:** Characterization of the participants

Long COVID patients (n=80 adults aged 30-50 years, n=80 adolescents aged 16-18 years) in whom subjectively perceived cognitive symptoms such as brain fog, concentration disorders and fatigue persist for more than four weeks after a PCR-confirmed SARS-CoV-2 infection, leading to restrictions in productive areas of life such as school, training and professional life, can participate in the study.

4.1.1 Inclusion criteria

- Adults (female, male, diverse) aged 30 to 50 inclusive
- Adolescents (female, male, diverse) aged 16 to 18 years inclusive
- cognitive symptoms consistent with Long COVID disease and persisting for at least four weeks after PCR-confirmed infection with SARS-CoV-2
- Access to a technical medium (e.g. PC, tablet, smartphone with internet access) that can be used to participate in one of the digital intervention variants
- Consent of the participant or legal guardian.

4.1.2 Exclusion criteria

- Age under 16 years, between 19-29 years and over 51 years,
- diagnosed with cognitive impairment before infection with SARS-CoV-2,
- previous SARS-CoV-2 was not confirmed by PCR test,
- no access to PC, tablet, smartphone with internet access,

- no consent to participate.

4.2 Recruitment

A total of n=160 participants (n=80 adolescents, n=80 adults) will be recruited for this study. The recruitment process will take place successively over a planned period of approximately three months.

n=80 adults (female, male, diverse) aged 30 to 50 years inclusive will be recruited via the already established online platform of the DEFEAT-Corona study (study register number DRKS00026007, ethics vote 9948_BO_K_2021). The successive recruitment strategy is based on questionnaire data on cognitive impairments, the collection of which has already been approved as part of the DEFEAT study. If subjects meet the participation criteria, they will be asked whether they would like to take part in the study.

In addition, n=80 adolescents (male, female, diverse) aged 16 to 18 years inclusive will be recruited. In order to enable recruitment analogous to the adult subjects, an amendment to the Corona-DEFEAT study will be submitted (see parallel submitted amendment with the same submission date as this study protocol). Here, not only the consent of the adolescent him/herself is obtained online, but also the consent of at least one legal guardian.

In order to increase awareness of our study, flyers will be distributed to registered pediatricians (or family doctors) and in the Long COVID outpatient clinics of the Hannover Medical School (MHH) and partner clinics in addition to the announcement via the Corona-DEFEAT portal. Information about the study is also available on the MHH website.

4.3 Information and consent

Information and education about screening for the study is provided digitally in a first step as part of the already approved study project (DEFEAT, ethics vote 9948_BO_K_2021) ("Information and consent in prospective observational study"). If participants are eligible for the study, they will be informed about it s aims and content (see appendix with information). Study participants are informed on the aim, purpose and procedure of the study and its various branches before signing their consent (in the case of minors after obtaining the consent of a parent or legal guardian) to participate in the study. Participants are informed that the study is of a pilot nature, that the treatment may be unsuccessful and that consent to participate can be withdrawn at any time. This information is provided in a personal interview (onsite or by telephone). The informed consent documents for adults and adolescents (Appendices #1-2 of this study protocol) and the informed consent forms (Appendices #3-4 of this study protocol) can be found in the Supplement of this application.

4.4 Participation and revocation of participation

Study participation is voluntary and can be terminated at any time point without providing reasons and without consequences. This can be done by not completing the digital survey instruments, by not attending therapy sessions or live online surveys, or by notifying us in writing or by telephone. Participants can request the deletion of data and information already collected at any time point by stating a request and their individually assigned pseudonym.

4.5 Study workflow - procedure and implementation

4.5.1 *Initial testing (t0)*

After consenting to participate in the study, participants will receive an invitation to baseline testing (time point 0; t0), which is performed to assess the status of subjective and objective cognitive and everyday life impairments associated with Long COVID. The following instruments are used to measure the effectiveness of the intervention for all participants:

1. COPM (Canadian Occupational Performance Measure) regarding productivity,
2. WIT-2 to test memory and cognitive work efficiency,
3. the IMET index to measure restrictions on participation,
4. the Neuro QoL to measure cognitive performance and fatigue symptoms.

All tests are described in detail in the appendix (see appendices #5-8 of this study protocol). In addition, sociodemographic and medical data will be collected at t0 (age, gender, type of education/training, occupation, time and duration of SARS-CoV-2 infection, severity of SARS-CoV-2 infection). The following analyses are to be carried out at t0:

The collection of analyses will be conducted online and carried out by qualified specialists (doctors, occupational therapists or employees who are trained and supervised by a psychologist (Prof. Dr. Christoph Berg, FOM University of Applied Sciences, Hamburg)). Testers are neither involved in treatment sessions during the intervention, nor do they know which group the participants are assigned to. The COPM is carried out within a 30-minute online appointment. The WIT-2 test in the areas of memory and cognitive work efficiency takes about 35 minutes. The following tests take place online and are completed independently by participants: IMET, NeuroQua_, EQ-5D-5L. Total test durations are between 10 and 20 minutes each, depending on the cognitive state. Tests should take place in one session (two sessions if the participants are too exhausted) after randomization at t0 and after the last therapy session at t1 (12 week time point) as well as at an online appointment at time t2 (24 week time point).

4.5.2 Randomization

After initial testing (t0), participants are assigned to three different groups (live online occupational therapy: n=20 adults, n=20 adolescents, video occupational therapy: n=20 adults, n=20 adolescents, no intervention: n=40 adults, n=40 adolescents). Randomization is performed employing digital tools (e.g. Randomizer: A Randomization (Randomization) Service for Multicenter Clinical Trials). Participants will be randomized into the following study arms:

1. Group A: n=20 adolescents and n=20 adults receive live online occupational therapy twice a week over a period of twelve weeks, each session lasting 30 minutes. This is carried out by specially trained occupational therapists.
2. Group B: n=20 adolescents and n=20 adults receive access to non-individualized occupational therapy video modules (video online occupational therapy) over a period of twelve weeks, two videos per week for self-application.
3. Group C: n=40 adolescents and n=40 adults receive no intervention over a period of twelve weeks and thus form the comparison group.

4.5.3 Control testing (t1 and t2)

Immediately after the twelve-week intervention (t1), as well as at a further point in time (24 weeks after inclusion (t2)), measurements are to be taken again using identical instruments as in the initial measurement (t0).

4.5.4 Data analysis of the non-blinded, randomized controlled pilot study

The evaluation of the pilot study will be conducted using a generalized linear mixed-effects model (GLME), a class of regression models. This model is used when the set of covariates analyzed to evaluate an intervention is not necessarily normally distributed and there is data with more than one source of random variability. In this case, planned repeated measures over time show likely variation within a respondent and between respondents. The model is intended to show the relationships between

1. measurable patient and therapist characteristics and probability of remission,
2. random variability in patient groups and in individual remission outcomes, and
3. between the different therapists and respective remission probabilities.

The GLME should help to make an informed decision about whether the individual differences in disease progression after intervention correlate with the digitally mediated occupational therapy or with individual factors of the intervention.

4.5.6 *Process evaluation using the realist evaluation technique*

In addition to the above-mentioned procedures, a process analysis will be carried out using realist evaluation, which focuses on evaluating the effectiveness and feasibility of the pilot study. According to Bastian et al. (2009), realist evaluation does not focus on linear cause-and-effect statements, but rather on the interplay of contexts through social interactions that make use of the intervention.

Based on this method, we aim to determine the effects and influences of the digital occupational therapy intervention (live online occupational therapy units or on-demand videos) on the health status of the study participants, as well as the feasibility and applicability of both types of interventions from the users' perspective.

To this end, an evaluation of the content will be carried out by the study participants in both treatment groups after each intervention module. An online questionnaire (Appendix #9) will be made available to participants at the end of each module via the online platform using the SoSci survey tool.

After completion of the intervention, additional qualitative expert interviews will be conducted with the occupational therapists and a subset of study participants in order to collect their perspectives with regard to applicability, acceptance, time required, and handling with the digital format and intervention. The study participants will also be asked about their perception of the effects of the intervention on concentration and improvement in everyday life. In addition, information on treatment adherence and reasons for discontinuation etc. will be determined.

The aim of this approach is to determine the forms of intervention in terms of their feasibility, appropriateness, and manageability. All occupational therapists providing treatment will be surveyed. Study participants are selected at random (e.g. via https://www.randomizer.at/). A total of 16 participants (n=16) were to be interviewed: Adolescents (n=8, n=4 of whom were female), in equal proportions from both intervention groups; adults (n=8, n=4 of whom were female) in equal proportions from both intervention groups.

4.6 Pseudonymization and data protection

Data protection is implemented in accordance with legal data protection regulations. Test subjects are informed about all essential data protection aspects and give their written consent after being informed. The data collected in this study is divided into four data sets:

1. Personal data (name, e-mail address),

2. socio-demographic data, clinical characterization (information on age, gender, migration background, health status, symptoms, etc.) as well as attitude towards vaccination and experience with COVID-19 vaccination,

3. test results (scores in cognitive tests, etc.),

and separately from this:

4. qualitative data (interview notes).

In order to protect it from misuse, data from the first three areas flows into two independent databases, which are stored separately from each other. Personal data (data set 1) is stored in encrypted form, physically separated from all other data collected (data sets 2 and 3).

The personal data and declarations of consent are stored exclusively at the respective study center. The online survey data is collected using the survey program SoSci Survey provided by Hannover Medical School. By default, this does not record IP addresses; the data is stored and processed on the server of the MHH data center in accordance with data protection regulations, in particular the European General Data Protection Regulation (GDPR). The data of the qualitative part of the study is stored on servers of the University Medical Center Göttingen. The data will be irretrievably destroyed ten years after the end of the study. Consent forms collected in paper form are stored in securely lockable filing cabinets. Only the respective study leaders have access. The scientific and treatment team has no access to the personal data. Data sets 2 and 3 are pseudonymized (pseudonymized ID, PID) using a combination of characters (5 digit number/ letter combination) so that it is not possible to draw conclusions about the identity of study participants. Processing and storage of the data as well as the entire data processing of all non-personal data is carried out using PIDs. A PID key allows for assignment of personal data (data set 1) to pseudonymized data (data sets 2 and 3). Only the respective study center has access to this key. This key is created by the recruiting study center upon inclusion in the study and passed on to the data trustee. This task lies with a designated person from the study team, who is likely to be the research group leader. No other persons can access it. The study center only has the PID key and has no direct access to data sets 2 and 3.

Re-identification, i.e. the linking of personal data and pseudonymized data sets, may be necessary in certain cases: The study participants order the destruction of data; the study participants have consented to being contacted again by the study team and follow-up examinations and/or the re-acquisition of data become necessary. There is imminent danger. In these exceptional cases or for similar or corresponding reasons, access to personal data is provided. Access can only be initiated by the project management. The following requirements must be met: The study team must have the subject's consent to be contacted repeatedly. The Ethics Committee of the Hannover Medical School must agree to the removal of the pseudonymization. Only under these conditions will the PID key, which enables the assignment of personal data and PID, be issued. This concept ensures that no employee can gain access to the clear name and study results at the same time.

The chosen procedure largely avoids collection of personal data (such as date of birth, address, etc.). The physical separation of consent documents and questionnaire and other data provides greatest possible security. Even in the unlikely event of data loss, retrograde identification of the subjects is unlikely. Study staff are obliged to comply with the applicable data protection and data security regulations.

Qualitative data (4) are processed exclusively at the University Medical Center Göttingen; other project members do not have access to them. After transcription of interview notes, transcripts are pseudonymized, other references (place of residence, names of doctors, etc.) that allow retrograde identification are deleted (*de facto* anonymization). Audio raw data/ video data are then destroyed. Transcripts are stored on digital data storage devices with password protection in a lockable filing cabinet. Transcripts will be deleted after ten years if the respondent does not agree to the *de facto* anonymized data being used for further research projects.

5. List of appendices

1. Patient information adolescents 16-18 years of age (including information for parents)
2. Informed consent for adults aged 30-50
3. Declaration of consent for adolescents aged 16-18 (incl. parental consent form)
4. Declaration of consent for adults aged 30-50
5. Information on the COPM test instrument (Canadian Occupational Performance Measure)
6. Information on the Wit2 test instrument
7. Information on the IMET test instrument for measuring restrictions on participation
8. Information on the Neuro QoL test instrument for cognitive performance and fatigue
9. Evaluation process questionnaire

6. Bibliography

Al-Aly, Z., Bowe, B. & Xie, Y. Long COVID after breakthrough SARS-CoV-2 infection. *Nature Medicine* (2022). https://doi.org:10.1038/s41591-022-01840-0

AOTA. (2021). *Information Pertaining to Occupational Therapy in the Era of Coronavirus (COVID-19).* Retrieved 04 28, 2022, from American Occupational Therapy Association: https://www.aota.org/Practice/ Health Wellness/COVID19.aspx.

Barkel, C., Lap, C. R., Buddingh, E. P., van Houten, M. A., van der Sande, L., Langereis, E., & Terheggen-Largro, S. (2021). Pediatric long-Covid: An overlooked phenomenon? *Pediatric pulmonology*, 2495-2502. doi:10.1002/ppul.25521

Beli, S., Babi, B., Price, I., Cattaneo, D., Masocco, F., Zaccaria, S., & Spruit, M. A. (2020). Low physical functioning and impaired performance of activities of daily life in COVID 19 patients who survived hospitalisation. *Eur.Respir J, 15*(56). doi:doi:10.1183/13993003.02096-2020

DVE, D. V. (2018). *Kompetenzprofil Ergotherapie.* Retrieved 04 28, 2022, from DVE: https://dve.info/resources/pdf/ergotherapie/kompetenzprofil-ergotherapie/3633-2019-kompetenzprofil/file

Hugon, J., Msika, E., Queneau, M., Farid, K., & Paquet, C. (2021). Long COVID: cognitive vomplaints (brain fog)and dysfunction of the cinglate cortes . *Journal of neurology*, 1-3.

Kassenärztliche Bundesvereinigung. (2021, 07 14). Retrieved from https://www.kbv.de/html/115_52751.php

Kataria, S., & Ravindran, V. (2018). Digital health: a new dimension in rheumatology patient care. *Rheumatol Int., 38*(11), 1949-1957. doi:10.1007/s00296-018-4037-x.

Kos, D., Duportail, M., Meirte, J., Meeus, M., D'hooghe, M., & Nagels, G. (2016). The effectiveness of a self-management occupational therapy intervention on activity performance in individuals with multiple sclerosis-related fatigue: a randomized-controlled trial. *International journal of rehabilitation research*.

Koczulla, A. R. *et al.* S1-Leitlinie Post-COVID /Long COVID. *Pneumologie* **75**, 869-900, doi:10.1055/a-1551-9734 (2021).

le Granse, M., van Hartingsveldt, M., & Kinébanian , A. (2017). *Grondslagen van de Ergotherapie.* Houten: Bohn Stafleu van Loghun.

Lopez-Leon, S., Wegman-Ostrosky, T., Perelman, C., Sepulveda, R., Rebolledo, P., Cuapio, A., & Villapol, S. (2021). More than 50 Long-term effects ov COVID-19: a systematic review and meta-analysis. *medRciv*. doi:10.1101/2021.01.27.21250617

Margetis, J. L., Wilcox, J., Thompson, C., & Mannion, N. (2021). Occupational therapy: Essential to critical care rehabilitation. *American Journal of occupational therapy, 75*(2), 7502170010p1-7502170010p5. doi:10.5014/ajot.2021.048827

Miller, F., Nguyen, V., Navaratnam, A., Shrotri, M., Kovar, J., Hayward, A., & Hardelid, P. (2021). Prevalence of persistent symptoms in children during the COVID-19 pandemic: evidence from a household cohort study in England and Wales. *medRxiv*. doi:10.1101/2021.05.28.21257602

NICE. (2022). *Rapid guideline: managing the long term effects of COVID-19.* Retrieved from https://www.nice.org.uk/guidance/ng188/resources/covid19-rapid-guideline-managing-the-longterm-effects-of-covid19-pdf-51035515742

Nalbandian, A. (2021) Post-acute COVID-19 syndrome. Nature Medicine. 27,601-615 doi:https://doi.org:10.1038/s41591-021-01283-z

Nittas, V., Gao, M., West, E. A., Ballouz, T., Menges, D., Wulf Hanson, S., & Puhan, M. A. (2022). Long COVID through a Public Health Lens: An Umbrella Review. *Public HEalth Reviews*. doi:10.3389/phrs.2022.1604501

Peine, A., Paffenholz, P., Martin, L., Dohmen, S., Marx, G., & Loosen, S. H. (2020). Telemedicine in Germany During the COVID-19 Pandemic: Multi-Professional National Survey. *J Med Internet Res, 22*(8), e19745. doi:10.2196/19745

Townsend, L., Dyer, A. H., & Jones, K. (2020). Persistent fatigue following SARS-CoV-2 Infection is common and independent of severity of initial infection. *Plos One, 15*(11), e0240784.

Walzer, S. (2022). Digital Healthcare in Germany, Contributions to Economics. Springer Nature. doi:https://doi.org/10.1007/978-3-030-94025-6_2

Wong-Chew,R.(2022). Symptom cluster analysis of long COVID-19 in patients discharged from the Temporary COVID-19 Hospital in Mexico City. Therapeutic Advances in Infectious Disesase doi:https://doi.org/10.1177/20499361211069264

7. A Appendices

Appendix 1: Information for adolescents from the age of 16 (including information for parents)

Head of studies
PD Dr. Alexandra Dopfer-Jablonka

Central contact site
PD Dr. med. Alexandra Dopfer-Jablonka
Klinik für Immunologie und Rheumatologie - OE 6830
Medizinische Hochschule Hannover
Carl Neuberg Straße 1
30625 Hannover
Email: Jablonka.Alexandra@mh-hannover.de
Tel: +49 511 532 3014

Information for young people aged 16 and over on the study: "ErgoLoCo" - online occupational therapy for Long COVID

Dear participating adolescents,

dear parents, dear legal guardians,

We invite you and your offspring to participate in the study for our "ErgoLoCo" model project on online occupational therapy. With this study, we aim to better understand and treat Long COVID in adolescents and adults.

In the following, we would like to inform you/you or your offspring about the objectives and the course of this study. We would also like to explain to you why participation is important. We ask you/your child to read this information carefully and then decide whether or not you wish to take part in the study.

Participation in this study is voluntary. Participation will only take place if you give your consent. If participation is refused or revoked now or later, you/your offspring will not suffer any disadvantages.

Since you are interested in the study, you probably already received some information about it.

The following text is intended to inform you once again about the most important aspects of the study - in particular about the objectives and the procedure. Please read this patient information carefully and conscientiously. A member of the study team will then conduct an informative discussion with you during a telephone or online appointment. Please do not hesitate to address any points that are unclear to you. You will be given sufficient time to consider whether you and/or your offspring should participate. For further information, you can contact our study team at any time (see contact details above).

Part I: Information about the study

1. What are the aims of the study?

The aim of the study is to show whether we can improve concentration problems that adolescents and adults with Long COVID suffer from by providing them with online occupational therapy (a form of movement/activity instruction) taught in videos or live online tutorials. At the moment, there are a lot of patients with concentration problems after COVID-19. Unfortunately, we don't have any good therapy options yet. We want to test whether online occupational therapy improves concentration problems in Long COVID. For this purpose, half of all participants will receive online occupational therapy sessions, the other half will receive no intervention as a control group. Which group you/your child falls into will be decided at random and communicated after the first tests.

2. What methods will be used and how will the study be conducted?

If the online tests or an examination by a supervising doctor gives rise to a reasonable suspicion that there may be concentration problems due to Long COVID, you and your parents will be informed about the study and asked for consent to participate.

The study team will provide you and your parents with this information and explain all study measures in a live online interview.

If you and your parents agree to participate in the study, you/your offspring will receive a participant identification number. All data for the study will then only be stored under this study number (pseudonymized).

If you agree to participate, we will arrange two online appointments to test your/your offspring's concentration and memory abilities, quality of life, and participation in everyday life. This allows us to assess how relevant the restrictions caused by Long COVID are in your/their everyday life and provide a baseline value to assess whether online occupational therapy will improve symptoms. For the tests and subsequent occupational therapy, you/your offspring will need an online device such as a personal computer or tablet with microphone and camera. The tests and the subsequent therapy are carried out exclusively by trained staff who have an obligation to professional secrecy, and sessions will be provided in a protected digital space. The interview and tests at the first appointment will last around 30 minutes.

This is followed by random assignment to the various study arms (randomization). It is decided at random whether you/your offspring will be in an intervention group or a control group.

While the control group (50% of all participants) receives no treatment and is tested again after twelve and 24 weeks for their ability to concentrate and quality of life (again by means of an online appointment, lasting approx. 30 minutes), the intervention groups (50% of participants) receive half-hour digital occupational therapy sessions twice a week from week zero to twelve. Here, 25% of all participants receive live online occupational therapy, i.e. they meet online with occupational therapists (Timm-Cook practice, Hannover) and are trained in techniques that make it easier for them to deal with concentration problems in everyday life. The other half of the participants in the intervention group (25% of all people who took part in the first test) receive digital occupational therapy using prerecorded occupational therapy videos specially tailored to then needs of Long COVID patients (also twice half an hour per week). These videos can be accessed "on-demand".

Both intervention groups also receive instructions for regular training in everyday life to help them cope better with the restrictions caused by Long COVID.

In addition to testing cognitive abilities and quality of life, we will ask you/your offspring to share your views on diagnosis, treatment, and physical and mental health. If you and your parents agree, we will provide questionnaires for you to answer in pseudonymized form (online or, if desired, in printed version).

All data will be analyzed pseudonymously, i.e. without directly identifying you as a participant. By analyzing the data, we want to ensure that Long COVID can be better treated in adolescents and adults in the future.

3. What are the risks for me if I participate?

Risks could theoretically arise from breach of data protection. This is explained in detail in part 2 of this information.

4. When am I not allowed to take part in the study?

The following persons are excluded from participation in the study:

- Age under 16 years, between 19-29 years and over 51 years,
- diagnosed cognitive impairment prior to infection with SARS-CoV2,
- previous SARS-CoV2 was not confirmed by PCR test,
- no access to PC, tablet, smartphone with internet access,
- no consent to participate.

5. Can I end the study prematurely?

Participation in the study can be terminated at any time point without giving reasons. Termination of participation will have no negative effects for you/your child.

6. What personal benefits do I gain and do I receive any compensation?

By participating, you/your child will have the chance to take part in a novel treatment trial for Long COVID. We take Long COVID seriously and hope that symptoms can be improved through a structured online intervention. But it must be clear to all participants that the study is of pilot and experimental nature. This means that we cannot say whether the digital intervention will improve symptoms. We also cannot say to what extent the type of therapy will be accepted by the patients. Participation could potentially be beneficial for individual study participants, but the overall aim of the study is to better understand treatment options for Long COVID in general and to improve care for all adolescents (young adults) and adults with this condition. It is not possible to predict whether study participants themselves will benefit from this. No compensation for your participation or expenses is planned.

7. What happens if the examination reveals incidental findings?

As the study may lead to results or findings that indicate another disease that is not part of the study, we ask you and your parents to inform us before the start of the study whether you wish to be made aware of these findings (see declaration of consent). Depending on the nature of possible incidental findings, this may result in further recommendations regarding therapy or further examinations.

8. Is there student/accident/travel insurance?

As the accompanying study purely takes place online, no study/accident or travel insurance exists.

Part II: Privacy policy

II. 1. What happens to my data?

For the purpose of conducting the clinical study, medical findings and personal information (such as age, gender, time of COVID-19 disease) about you/your offspring will be collected and documented by study staff in the study database.

The data important for the clinical study will only be stored in encrypted (pseudonymized) form in a password protected, electronical database. Pseudonymized means that no names or initials are used, only a number or letter code. The data is protected against unauthorized access. Only on site study management and their immediate team will be able to identify you/your offspring personally on the basis of the encrypted data. The study data will be passed on to the study center in pseudonymized form. The study data will only be passed on to third parties in anonymized form; this means that it is no longer possible to identify you/your child.

Your/your offspring ´s name and date of birth will be entered in the consent form, which will be stored separately from all other study data. It is possible that inspectors from official monitoring authorities may inspect these documents in order to check that the study is being conducted in accordance with legal regulations. Inspectors are professionally obliged to treat your (your/your offspring´s) personal data confidentially.

The legal basis for the processing of personal data is your voluntary written consent in accordance with the GDPR (Art. 6 § 1 lit. a) in conjunction with Art. 9 § 2 lit. a)) for the processing of sensitive data. Your consent is voluntary and can be withdrawn at any time point and without any adverse effects. Without your consent to the processing and disclosure of relevant data in encrypted form, you/your offspring cannot participate in the above mentioned clinical study. Publications in journals and public trial registries (e.g. clinicaltrials.gov or EU Clinical Trials Registry) or presentations of trial results will not contain any data that can be used to personally identify your offspring.

Your data/your offspring´s data will be processed in this study primarily for the above-mentioned purpose, namely the improvement of diagnosis and therapy in adolescents and adults with Long COVID. However, it is possible that in the course of the investigation and data analysis, further research questions may arise that are related to the subject of this study. In this case, your data/your child's data would also be used for this purpose. However, you can explicitly object to this in the consent form. Your/your offspring 's data will be stored by the study center until 31.12.2023. After that, your child's data will be completely anonymized or deleted. After anonymization or deletion, it is no longer possible to draw conclusions back to you/ your offspring.

Responsible for data processing in the context of this clinical study is

Prof. Georg Behrens, Clinic for Rheumatology and Immunology, Carl-Neuberg-Str. 1, 30625 Hannover, Germany

With regard to the data, you have the following rights that you can assert against the controller:

Right to information: You have the right to information about the personal data concerning you/your offspring that is collected, processed or, if applicable, transmitted to third parties as part of the clinical trial (including a free copy). You may also request the provision of a portable electronic data carrier on which the data concerning your child is stored in a structured and commonly used format (Office or PDF file) or the transfer of this data to another controller* (Article 15 GDPR).

Right to deletion: You have the right to deletion of personal data concerning you/your offspring child, e.g. if this data is no longer required for the purpose for which it was collected (Article 17 GDPR).

Right to restriction of processing: Under certain conditions, you have the right to request a restriction of processing, i.e. the data may only be stored but not processed. You must request this (Article 18 GDPR).

Right to data portability: You have the right to receive the personal data concerning you/your offspring that you have provided to the* controller* for the clinical trial. This enables you to request that this data (structured and in a commonly used format on a portable electronic medium) to be transmitted either to you or to another (further) controller designated by you for data processing within the meaning of the GDPR (Article 20 GDPR).

Right to object: You have the right to object at any time to specific decisions or measures concerning the processing of personal data relating to you/your offspring. Processing (of new data) will then no longer take place unless processing is still required by law - as in the German Medicinal Products Act (AMG) (Article 21 GDPR). If you would like to exercise these rights, please contact the study investigators or the data protection officer of your trial center.

Restrictions: We would like to take this opportunity to point out that the rights listed may be restricted if these rights make it impossible or seriously impair the realization of the research purposes and the restriction is necessary for the fulfillment of the research purposes (Article 89 GDPR, §27 BDSG-new). Youw/your offspring´s rights to information, data portability and rectification of incorrectly processed data do not exist if the provision of information would require a disproportionate effort or is technically impossible. Whether your/your offspring´s rights can be restricted requires a specific assessment.

You have the **right to file a complaint** with a supervisory authority if you consider that the processing of personal data relating to you infringes the GDPR.

Data Protection Officer* / Data Protection Supervisory Authority

State Commissioner for Data Protection of Lower Saxony (LfD)

P.O. Box 221

30002 Hannover

Tel.: 0511 120-4500, Fax: 0511 120-4599

E-Mail: poststelle@lfd.niedersachsen.de

Data Protection Officer of Hannover Medical School

Carl-Neuberg-Str. 1

30625 Hannover

Tel.: 0511- 532-2555

E-Mail: Datenschutz@mh-hannover.de

____

(Date, name & signature of principal investigator or investigator)

____

(Date, name & signature of participating adolescent

____

(Date, name & signature of legal guardian

Appendix 2: Information for adults

Head of studies
PD Dr. Alexandra Dopfer-Jablonka

Central contact site
PD Dr. med. Alexandra Dopfer-Jablonka
Klinik für Immunologie und Rheumatologie - OE 6830
Medizinische Hochschule Hannover
Carl Neuberg Straße 1
30625 Hannover
Email: Jablonka.Alexandra@mh-hannover.de
Tel: +49 511 532 3014

Information for adults on the study: "ErgoLoCo" - online occupational therapy for Long COVID

Dear participants,

We would like to invite you to take part in the study for our "ErgoLoCo" pilot project on online occupational therapy. In the study, we are trying to better understand and treat Long COVID in adolescents and adults.

In the following, we would like to inform you about the objectives and the course of this study. We would also like to explain why participation is important. We ask you to read this information carefully and then decide whether or not you wish to participate in the study.

Participation in this study is voluntary. Participation will only take place if you give your consent. If participation is refused or revoked now or later, you will not suffer any disadvantages.

Since you are interested in the study, you probably already have some information about it. The following text is intended to inform you about the most important aspects of the study - in particular the objectives and the procedure.

Please read the participation information carefully and conscientiously. A member of the study team will then conduct an informative discussion with you during an online appointment. Please do not hesitate to raise any points that are unclear to you. You will be given sufficient time to think about your participation. For further information, you can contact our study team at any time point (please see contact details above).

Part I: Information about the study

1. What are the aims of the study?

The study aims to test whether we can improve concentration problems experienced by adolescents and adults with Long COVID by providing them with online occupational therapy (a form of movement/activity instruction) delivered in videos or live online tutorials.

At the moment, we are seeing a surge of patients suffering from concentration problems after COVID-19. Unfortunately, we do not have any good therapy options yet. We want to test whether online occupational therapy improves concentration problems in Long COVID. To this end, half of all participants will receive online occupational therapy sessions, while the other half will receive no intervention and serve as a control group. Which group you will be assigned to will be decided at random and communicated after the first tests.

2. What methods will be used and how will the study be conducted?

If the online tests or an examination by a supervising doctor gives rise to a reasonable suspicion that you may have concentration problems related to Long COVID, you will be informed about the study and asked for your consent to participate.

The study team will provide you with this information and explain all study measures in a live online interview.

If you agree to participate in the study, you will be given a study number. All data for the study will then only be stored under this study number (pseudonymized).

If you agree to participate, we will arrange an online appointment to test your concentration and memory abilities, quality of life, and participation in everyday life. This allows us to assess how severe the restrictions caused by Long COVID are in your everyday life and provides a baseline to estimate whether online occupational therapy improves your symptoms. For the tests and the subsequent occupational therapy, you will need an online device such as a personal computer or tablet with microphone and camera. The tests and subsequent therapy sessions are carried out exclusively by trained staff who are obliged to professional secrecy and will be performed in a protected digital space. Interview and tests at the first appointment will take about 30 minutes.

You will then be randomly assigned to the different study arms (randomization). A random decision is made as to whether you are assigned to an intervention or control group.

While the control group (50% of all participants) receives no treatment and is tested again after twelve and 24 weeks for their ability to concentrate and their quality of life (again by means of an online appointment lasting around 35 minutes), the interventional groups (50% of participants) receive half-hour digital occupational therapy sessions twice a week from week zero to week twelve. Here, 25% of all participants receive live online occupational therapy. In these sessions, they will meet online with occupational therapists (Timm-Cook practice, Hannover) and will be trained in techniques to facilitate dealing with concentration and occupational problems in everyday life.

The other half of the participants in the interventional group (25% of all those who took part in the first test) receive digital occupational therapy using prerecorded therapy videos specifically tailored to the needs of Long COVID patients (also twice weekly in half hour long sessions). Videos can be accessed "on-demand".

Both interventional groups also receive instructions for regular training in everyday life to help them cope better with the restrictions caused by Long COVID.

In addition to testing cognitive abilities and quality of life, we may ask you to share your views on diagnosis, treatment, and physical and mental health. If you agree, we will hand out questionnaires in pseudonymized form for you to answer (online or, if you wish, as in printed version).

All data will be analyzed pseudonymously, i.e. without direct reference to the person taking part.

By analyzing this data, we want to answer the question how Long COVID can be better treated in adolescents and adults in the future.

3. What are the risks for me if I participate?

Risks could theoretically arise from breaches in data protection. This is explained in detail in part 2 of this information.

4. When am I not allowed to take part in the study?

The following persons are excluded from participation in the study:

- Age under 16 years, between 19-29 years and over 51 years,
- diagnosed cognitive impairment prior to infection with SARS-CoV2,
- previous SARS-CoV2 was not confirmed by PCR test,
- no access to PC, tablet, smartphone with internet access,
- No consent to participate.

5 Can I end the study prematurely?

Participation in the study can be terminated at any time without providing reasons. Termination of participation has no negative effects for you.

6. What personal benefits do I gain and do I receive any compensation?

By participating, you have the chance to take part in a novel treatment trial for Long COVID. We take Long COVID seriously and hope that symptoms can be improved through a structured online intervention. But it must be clear to all participants that the study is of pilot and experimental nature. As such we cannot say whether the digital intervention will improve the symptoms of Long COVID. We also cannot say to what extent which type of therapy will be accepted by the patients. Participation could potentially be beneficial for individual study participants, but the overall aim of the study is to better understand treatment options for Long COVID in general and to improve care for adolescents and adults with this condition in general. It is not possible to predict whether study participants themselves will benefit from participation. No compensation for your participation and expenses is planned.

7. What happens if the examination reveals incidental findings?

As the study may produce results or findings that indicate another disease that is not part of the study, we ask you to inform us before the start of the study whether you wish to be made aware of such findings (see declaration of consent). Depending on the nature of possible incidental findings, this may result in further recommendations regarding therapy or further examinations.

8. Is there student/accident/travel insurance??

As this study only takes place online, no study/accident or travel insurance exists.

Part II: Privacy policy

II. 1. What happens to my data?

For the purpose of conducting the clinical trial, medical findings and personal information (such as age, gender, time of COVID-19 disease) about you will be collected and documented by study staff in the study database.

The data important for the clinical trial will only be stored in encrypted (pseudonymized) form in a password-protected, electronic database. Pseudonymized means that no names or initials are used, only a number or letter code. The data is protected against unauthorized access. Only the study management on site and their team will be able to identify you personally using the encrypted data. The study data will be passed on to the study center in pseudonymized form. The study data will only be passed on to third parties in anonymized form; this means that it is no longer possible to identify you.

Your name and date of birth will be entered on the consent form, which will be stored separately from all other study data. It is possible that inspectors from official monitoring authorities may inspect these documents in order to check that the study is being conducted in accordance with legal regulations. Inspectors are professionally obliged to treat your personal data confidentially.

The legal basis for the processing of the personal data concerned is your voluntary written consent in accordance with the European General Data Protection Regulation (GDPR, Art. 6 §1 lit. a), Art. 9 §2 lit. a)) for the processing of sensitive data. Your consent is voluntary and can be withdrawn at any time point without any adverse effects. Without your consent to the processing and disclosure of the relevant data in encrypted form, you cannot participate in the above mentioned clinical study. Publications in journals and public trial registries (e.g. clinicaltrials.gov or EU Clinical Trials Registry) or presentations of trial results will not contain any data that can be used to identify you personally.

Your data will be processed in this study primarily for the above mentioned study purpose, namely the improvement of diagnosis and therapy in adults and adolescents with Long COVID. However, it is possible that in the course of the investigation and data analysis, further research questions may arise that are related to the subject of this study. In this case, your data would also be used for this purpose. However, you can explicitly object to this in the consent form.

Your collected data will be stored by the study center until 31.12.2023. After that, it will be completely anonymized or deleted. After anonymization or deletion, it is no longer possible to draw conclusions back to you.

Responsible for data processing in the context of this clinical study is

Prof. Georg Behrens, Clinic for Rheumatology and Immunology, Carl-Neuberg-Str. 1, 30625 Hannover, Germany

II. 3. Supplementary information according to the European Data Protection Basic Regulation

You have the following rights with regard to the data, which you can assert against the controller:

Right to information: You have the right to information about the personal data concerning you that is collected, processed or, if applicable, transmitted to third parties in the context of the clinical study (including a free copy). You may also request the provision of a portable electronic data carrier on which the data concerning you are stored in a structured and commonly used format (Office or PDF file) or the transmission of these data to another controller* (Article 15 GDPR).

Right to deletion: You have the right to deletion of your personal data, e.g. if this data is no longer required for the purpose for which it was collected (Article 17 GDPR).

Right to restriction of processing: Under certain conditions, you have the right to request a restriction of processing, i.e. the data may only be stored but not processed. You must request this (Article 18 GDPR).

Right to data portability: You have the right to receive the personal data concerning you that you have provided to the trial investigators. This means that you can request that this data (structured and in a commonly used format on a portable electronic data carrier) can be transmitted either to you or to another (further) controller designated by you for data processing within the meaning of the GDPR (Article 20 GDPR).

Right to object: You have the right to object at any time point to specific decisions or measures relating to the processing of personal data concerning you. Processing (of new data) will then no longer take place unless processing is still required by law - as in the German Medicinal Products Act (AMG) (Article 21 GDPR). If you would like to exercise these rights, please contact the study team or the data protection officer of your trial center.

Restrictions: We would like to take this opportunity to point out that the rights listed may be restricted if these rights make it impossible or seriously impair the realization of the research purposes and the restriction is necessary for the fulfillment of the research purposes (Article 89 GDPR, §27 BDSG-new). Your rights to information, data portability and rectification of incorrectly processed data do not exist if the provision of information would require a disproportionate effort or is technically impossible. Whether your rights can be restricted requires a specific assessment.

You have **the right to lodge a complaint** with a supervisory authority if you consider that the processing of personal data relating to you infringes the GDPR.

Data protection officer/ data protection supervisory authority

State Commissioner for Data Protection of Lower Saxony (LfD)

Postfach 221

30002 Hannover

Tel.: 0511 120-4500, Fax: 0511 120-4599

E-Mail: poststelle@lfd.niedersachsen.de

Data Protection Officer of the Hannover Medical School

Carl-Neuberg-Str. 1

30625 Hannover

Tel.: 0511- 532-2555

E-Mail: Datenschutz@mh-hannover.de

____

(Date, name & signature of principal investigator or investigator)

____

(Date, name & signature of participant)

Appendix 3: Declaration of consent for adolescents aged 16 and over (incl. parental consent form)

Head of studies
PD Dr. Alexandra Dopfer-Jablonka

Central contact site
PD Dr. med. Alexandra Dopfer-Jablonka
Klinik für Immunologie und Rheumatologie - OE 6830
Medizinische Hochschule Hannover
Carl Neuberg Straße 1
30625 Hannover
Email: Jablonka.Alexandra@mh-hannover.de
Tel: +49 511 532 3014

Consent for adolescents aged 16 and over for the study: "ErgoLoCo" - online occupational therapy for Long COVID

PART A: Consent of adolescents

PART B: Consent of legal guardians

PART A: to be signed by the adolescent

I have read and taken note of the information about the study, including the privacy policy. Any questions I may have had were answered satisfactorily by the person responsible for the study and I have had sufficient time to consider my participation.

....	....	I_I_I I_I_I I_I_I_I_I
First name of participant	Last name of participant	Date of birth

I give my consent to the points marked below:

- □ Participation in the study with the knowledge that participation in the study can be terminated by me at any time.
- □ Processing of my data only for study purposes and purposes related to the study objective.
- □ To receive invitations to questionnaires and to arrange further study appointments if necessary.

If medically relevant incidental findings arise in the course of the examination or during the evaluation of my data, I would like the following to happen:

- □ I wish to be informed of any incidental findings.
- □ I wish not to be informed of any incidental findings.

In the event of revocation of my consent to participate in the study:

- □ May all my data collected thus far be used for the purposes of this study and study purposes and purposes related to the study objective.
- □ May my previously collected data be used for the purposes of this study.
- □ Must all my no longer required data be deleted immediately.

My consent to participate in the study is voluntary and I can revoke it at any time for the future without providing reasons. The withdrawal of consent shall not affect the lawfulness of processing based on consent before its withdrawal.

____

(Date, name & signature of investigator)

____

(Date, name & signature of the 17-18 year old participant)

Last name, first name: ____

Study ID of the participant:
to be filled in by the doctor

-- -- -- --	-- -- -- --	-- -- -- --	-- -- -

Part B: to be signed by the legal guardian(s)

I have read and taken note of the information about the study, including the privacy policy. Any questions I had were answered satisfactorily by the person responsible for the study and I had sufficient time to consider my child's participation in the project.

....	....	I_I_I I_I_I I_I_I_I_I
First name of the participant	Last name of the participant	Date of birth

In the following I give my consent to the points selected:

- □ Participation of my offspring in the study with the knowledge that participation in the study can be terminated by me at any time.

□ Processing of the data collected during my offspring´s participation only for study purposes and purposes related to the study objective.

□ For my offspring to receive invitations to questionnaires and to arrange further study appointments if necessary.

If medically relevant incidental findings are discovered in the course of the examination or during the evaluation of my offspring´s data, I would like the following to happen:

□ I or my offspring should be informed of any incidental findings.

□ I or my offspring should be not informed of any incidental findings.

In the case of a withdrawal of my consent or the withdrawal of consent by my offspring to participate in the study:

□ May all of my offspring´s data collected so far be used for the purposes of this study and study purposes and purposes related to the study objective.

□ May my offspring`s data collected thus far be used for the purposes of this study.

□ Must all my offspring´s no longer required data be deleted immediately.

Consent to participate in the study is voluntary and I or my offspring can revoke it at any time for the future without providing reasons. The withdrawal of consent shall not affect the lawfulness of processing based on consent before its withdrawal.

____

(Date, name & signature of investigator)

____

(Date, name & signature of the legal guardian)

Last name, first name: ____

Study ID of the participant:
to be filled in by the doctor

-- -- -- --	-- -- -- --	-- -- -- --	-- -- -- -

Appendix 4: Declaration of consent for adults

Head of studies
PD Dr. Alexandra Dopfer-Jablonka

Central contact site
PD Dr. med. Alexandra Dopfer-Jablonka
Klinik für Immunologie und Rheumatologie - OE 6830
Medizinische Hochschule Hannover
Carl Neuberg Straße 1
30625 Hannover
Email: Jablonka.Alexandra@mh-hannover.de
Tel: +49 511 532 3014

Consent for adults for the study: "ErgoLoCo" - online occupational therapy for Long COVID

I have read and taken note of the information about the study, including the privacy policy. Any questions I had were answered satisfactorily by the person responsible for the study and I had sufficient time to consider my participation in the project.

....	....	I_I_I I_I_I I_I_I_I_I
First name of participant	Last name of participant	Date of birth

In the following I give my consent to the points ticked:

- □ Participation in the study with the knowledge that participation in the study can be terminated by me at any time.
- □ Processing of my data only for study purposes and purposes related to the study objective.
- □ To receive invitations to questionnaires and to arrange further study appointments if necessary.

If medically relevant incidental findings arise in the course of the examination or during the evaluation of my data, I would like the following to happen:

- □ I wish to be informed of any incidental findings.
- □ I wish not to be informed of any incidental findings.

In the event of revocation of my consent to participate in the study:

- □ May all my data collected thus far be used for the purposes of this study and study purposes and purposes related to the study objective.
- □ May my previously collected data be used for the purposes of this study.
- □ Must all my longer required data be deleted immediately.

My consent to participate in the study is voluntary and I can revoke it at any time for the future without providing reasons. The withdrawal of consent shall not affect the lawfulness of processing based on consent before its withdrawal.

____

(Date, name & signature of investigator)

____

(Date, name & signature of the participant)

Last name, first name: ____

Study ID of the participant:
to be filled in by the doctor

-- -- -- --	-- -- -- --	-- -- -- --	-- -- -- --

Appendix 5: Information on the COPM test instrument

The COPM (Canadian Occupational Performance Measure) test records self-assessment of occupational performance and satisfaction in a structured manner. The test is carried out online by the test subjects under guidance of a qualified study employee. The duration is approx. 30 minutes. Here is an overview of the data collected:

Appendix 6: Information on the WIT-2 test instrument

The Wilde Intelligence Test 2 is an instrument to assess general intelligence with specific reference to professional work. The WIT-2 consists of eleven modules focusing on distinct facets of intelligence and work behavior. Two of these modules are used in the study. Module "MF" measures memory and consists of a memorization and a recall phase. Between these two phases, the "EM" module is used as a distractor to record cognitive work efficiency. For testing, the commercial "Hogrefe" test system will be employed and testing will be carried out online under the guidance of a qualified study supervisor. The duration of the test, including the instructions, is approx. 35 minutes.

Appendix 7: Information on the IMET test instrument

The IMET (Index for Measuring Restrictions on Participation) measures restrictions on participation in everyday life and is automatically collected via the Corona-Defeat Portal. The test is carried out online by the respondents themselves using a ready-made click questionnaire. The duration is approx. 10 minutes.

Appendix 8: Information on the NeuroQuoL test instrument

The Neuro QoL survey collects data on cognitive performance and fatigue and quality of life. The Neuro QoL is available in German. The test is carried out online by the respondents. The duration is approx. 5-10 minutes

Appendix 9: Questions on process evaluation

How were you able to follow the content of the last module (last four therapy sessions)?

☐ not at all ☐ rather not ☐ somewhat ☐ rather good ☐ good

Were you able to apply the contents of the last module in your everyday life?

☐ not at all ☐ rather not ☐ somewhat ☐ rather good ☐ good

Has your action problem changed within the last module?

☐ worsened ☐ rather worsened ☐ remained the same ☐ rather improved ☐ improved

What was particularly helpful for you in the last module?

Free text box

Did any problems (of any kind) occur during the last module?

Free text box

How satisfied were you with the last module?

☐ not satisfied at all ☐ Rather not satisfied ☐ neutral ☐ Rather satisfied ☐ satisfied

Zentrum für Innere Medizin
Klinik für Rheumatologie und Immunologie

OE 6830
PD Dr. med Alexandra Jablonka
Tel.: 0511 532- 5337
0170 - 3805936
Fax: 0511 532-8055
jablonka.alexandra@mh-hannover.de

Carl-Neuberg-Straße 1
30625 Hannover
Telefon: 0511 532-0
www.mh-hannover.de

MHH, Zentrum für Innere Medizin, OE 6830, 30625 Hannover

To the
Ethics committee of MHH
Prof. Dr. med. Bernhard Schmidt
OE 9515

9. Mai 2024

_

Amendment to the proposal DEFEnse Against COVID-19 Study – Looking forward: DEFEAT Corona 9948_BO_K_2021; Amendement regarding the subproject ErgoLoCo

Dear Professor Schmidt
Dear members of the Ethics Committee,

We would like to make an addition to our DEFEnse Against COVID-19 STudy - Looking forward, which we would be very grateful for your approval:
As part of our study and recruitment, we have noticed that the recruitment of children and adolescents is very difficult. We have already carried out extensive, Germany-wide advertising via social media, schools, pediatricians and Long COVID outpatient clinics. Since, contrary to our initial assumption, the children and adolescents affected by Long COVID suffer more from fatigue, but report only relatively minor impairments in the areas of cognition and memory, there are very few adolescents whose complaints match our recruitment scheme.
In addition, the children and adolescents are excellently cared for in terms of occupational therapy; around 90% have already undergone occupational therapy interventions or are planning to do so. We also have a high dropout rate in the control group in particular, as the children receive occupational therapy outside of the study and do not want to wait the planned waiting period. This is of course desirable with regard to the care of this patient group, but jeopardizes our study objective.
Due to the short recruitment period, we would like to slightly change the study design and include young adults in the group of children and adolescents, who, according to the occupational therapists, are very similar to adolescents in terms of their experiences. We would like to recruit adolescents and young adults between the ages of 16 and 25 (previously 16-18). The other study purposes and the design will not change. The participant information or consent forms also do not need to be adapted, as the previous documents cover all age groups from 16 years onwards. The number of subjects in the groups and previously approved subjects per group would also not be affected by this change.
To summarize, we would like to request that the two recruitment groups of previously adolescents (16-18 years) and adults (30-50 years) be changed to: Teenagers and Young Adults (16-25 years) and Adults (30-50 years).
Your approval would allow us to complete the project on time and maintain the validity and power of the study. It also better reflects the care needs of Long COVID. Please do not hesitate to contact us if you have any questions.
Yours sincerely

Alexandra Dopfer-Jablonka
Study Director

Zentrum für Innere Medizin
Klinik für Rheumatologie und Immunologie

OE 6830
PD Dr. med Alexandra Jablonka
Tel.: 0511 532- 5337
0170 - 3805936
Fax: 0511 532-8055
jablonka.alexandra@mh-hannover.de

Carl-Neuberg-Straße 1
30625 Hannover
Telefon: 0511 532-0
www.mh-hannover.de

MHH, Zentrum für Innere Medizin, OE 6830, 30625 Hannover

To the
Ethics Committee
Prof. Dr. med. Bernhard Schmidt
OE 9515

9. Mai 2024

–

Amendment to the proposal DEFEnse Against COVID-19 Study – Looking forward: DEFEAT Corona 9948_BO_K_2021; Amendement regarding the subproject ErgoLoCo

Dear Professor Schmidt
Dear members of the Ethics Committee,

We would like to make an addition to our DEFEnse Against COVID-19 STudy - Looking forward, which we would be very grateful for your approval:
As part of our study and recruitment, we have noticed that a high proportion of the subjects seeking help from us due to Long-COVID have not been PCR-detected for SARS-CoV-2 infection. After many years of the pandemic, many infections were only detected using rapid antigen tests or antibody detection.
We would therefore like to adapt our recruitment strategy and extend the inclusion criterion "post-weighted corona infection" to include the detection of SARS-CoV-2 infection by PCR, rapid antigen test or antibody test. The adjustment better reflects the test reality in long COVID patients. This explicitly does not change the study objective, group sizes or other factors in our study.
Your consent would enable us to tailor the project even better to the needs of those affected, complete it on time and maintain the significance and power of the study. Please do not hesitate to contact us if you have any questions.
Yours sincerely

Alexandra Dopfer-Jablonka
Study Director

Zentrum für Innere Medizin
Klinik für Rheumatologie und Immunologie

OE 6830
PD Dr. med Alexandra Jablonka
Tel.: 0511 532- 5337
0170 - 3805936
Fax: 0511 532-8055
jablonka.alexandra@mh-hannover.de

Carl-Neuberg-Straße 1
30625 Hannover
Telefon: 0511 532-0
www.mh-hannover.de

MHH, Zentrum für Innere Medizin, OE 6830, 30625 Hannover

To the
Ethics Committee
Prof. Dr. med. Bernhard Schmidt
OE 9515

9. Mai 2024

_

Amendment to the proposal DEFEnse Against COVID-19 Study – Looking forward: DEFEAT Corona 9948_BO_K_2021; Amendement regarding the subproject ErgoLoCo

Dear Professor Schmidt
Dear members of the Ethics Committee,

We would like to make a further addition to our DEFEnse Against COVID-19 STudy - Looking forward, and we would be very grateful for your approval:

As part of our study and recruitment, we have noticed that a high proportion of the subjects who seek help from us due to Long COVID are between 30 and 40 years old. The median age of patients who want to take part in our study is around 35.

We would therefore like to adapt our recruitment and evaluation strategy and change the group distribution so that we include n=80 subjects between 16 and 35 years (previously n=80 subjects between 16 and 30 years) and we include n=80 subjects between 35 and 70 years (previously n=80 subjects between 30 and 50 years).
The adjustment better reflects the care needs for Long COVID. Your approval would enable us to tailor the project even better to the needs of those affected, complete it on time and maintain the significance and power of the study. Please do not hesitate to contact us if you have any questions.
Yours sincerely

Alexandra Dopfer-Jablonka

Study Director
