# Supplementary material for: Improvement in quality of life and cognitive function in Post-COVID syndrome after online occupational therapy: Results from a randomized controlled pilot study
Source: PLoS One. 2025 May 20;20(5):e0312714. doi: 10.1371/journal.pone.0312714 (PMC12091760; doi:10.1371/journal.pone.0312714)
Supplement: Attachment — Submitted filename: ErgoLoCo PLOS ONE_Rebuttal.docx [file pone.0312714.s005.docx]

**REVIEWER COMMENTS:** Article Submission to PLOS Medicine PMEDICINE-D-24-01416R1 - [EMID:796be22ac9d02dab]

Reviewer #1: Authors conducted an unblinded randomized pilot study in order to test the feasibility and efficacy of an online occupational therapy compared to a control group in post-COVID patients. There were two experimental groups (interactive online treatment and prerecorded video sessions) and a control group. Results revealed significant improvements in cognition, quality of life and occupational performance for the interactive group compared to the other groups. Regarding feasibility, the interactive group reported greater positive experience compared to the video group.
The study is timely, however there are some main aspects that need to be addressed:

*1)* ***The aim of the study*** *needs clarification. The authors indicate "we tested the feasibility and efficacy of structured online OT for PCS in a randomized controlled, unpowered pilot study". Authors should specify efficacy on which aspects; Also, "The aim of the intervention was to support affected people in developing coping strategies against major PCS symptoms such as fatigue and cognitive impairments and thus improve their quality of life and social participation."
The aim needs clarification too: The aim was to support patients? Or to improve their coping strategies? how did the authors evaluate that the patients developed better coping strategies? Authors should operationalize the objectives. In the "outcomes section", authors specify as secondary objective "cognitive function", however, this is not reflected in the objectives of the study. The objectives and outcomes section should be aligned.*

We thank the reviewer for this valuable comment and aimed at aligning the description of the study objectives and aims with that of the outcomes. We specified the respective paragraph in the methods section.

*2)      Authors did not describe any exclusion criteria. Exclusion criteria should be specified.*

We now added these to the methods section.

*3)      The intervention is not described in dept, needs more detail. How long lasted the sessions? How many days between sessions? How the sessions were structured to include: 1) exercises to control "typical PCS symptoms" (please describe which symptoms were addressed and which exercises were performed); 2) customizable units to improve everyday occupations; 3) social participation; 4) wellbeing. How many sessions were focused on each objective? It would be helpful to include references regarding the intervention program used.*

We thank the reviewer for this comment and were happy to describe our intervention in more detail. We changed two respective paragraphs in the methods section.

*4)      Based on the secondary objectives of the study, did the authors include any cognitive intervention? If not, which is the rationale to expect cognitive changes after an intervention focused on exercises to control typical PCS symptoms, customizable units to improve everyday occupations, social participation and wellbeing?*

We thank the reviewer for this comment and added the respective information in the methods section of the revised manuscript. Within the occupational therapy intervention, there are exercises, training, and aids across several modules that can potentially improve cognition, either through direct training or by tapping into personal resources through better time or energy management. Some examples are listed here: Module 2 includes exercises for fatigue management, learning active/passive break integration, as well as mindfulness and breathing exercises. Module 3 includes, among other things, aids for learning to cope with stress. It also covers cognitive daily life training, such as planning activities or learning vocabulary, which also corresponds to memory training. A key focus of Module 4 is to achieve optimal use of one's remaining resources through the adaptation of the personal environment. This module also includes exercises for training cognitive daily life skills, such as orientation, long- and short-term memory training, spatial awareness, and attention. For more detailed information, please refer to the study protocol.

*5)      What do authors mean by "control" typical PCS symptoms?*

People with PCS are often times overwhelmed by everyday tasks due to lack of energy, concentration and attacks of fatigue. Our OT program aims at sensitizing PCS people for their personal abilities and sustainability factors (e.g. through pacing, organizing their personal day, relaxation strategies), thus “controlling” their symptoms to the degree that seems possible to them personally. We aimed at describing this clearly in the revised manuscript.

*6)      One of the main limitations of the study is the baseline differences between groups that could have influenced the results. The control group was older had had poorer memory performance and there were also sex differences, which is directly related with the objectives of the study. How did the authors control for it? Would the results be significant after controlling for age, sex, and baseline memory?*

The presented results are baseline adjusted for memory function, but not age and sex. We carefully revised all statistical methods, as reviewer #3 also provided valuable further suggestions, and present adjusted and unadjusted baseline and longitudinal data in the revised main body and supplement of the manuscript.

*7)      Years of education is a variable that directly influences cognition, and should reported for each group. Were there any significant differences in years of education between groups? If so, it should be included as covariate too.*

Thank you for this comment. Unfortunately, we do not have detailed information on the years of education for each proband and were unable to correct for this.

*8)      Results section: I would suggest the authors to clearly differentiate the paired-t-test results and the repeated measures results.*

Thank you for this suggestion. We structured the results with the following subheading to differentiate the results more clearly: Sample characteristics, longitudinal differences within groups and differences between groups

*9)      Discussion: I would suggest to compare present results with previous studies that also performed interventions with PCS to ameliorate symptoms (for example: Oliver-Mas et al., 2023).*

Thank you for this comment, we now added a part on novel non-pharmaceutical interventions for PCS treatment to the discussion.

*10)     Introduction and Discussion: These cognitive deficits in PCS are related to brain alterations, both structural and functional. I would suggest to include brief information regarding this associations, to contextualize and highlight the relevance of interventions in these patients.*

Thank you for this comment, we now added this information to the discussion.

*Minor points.
1) authors should specify abbreviations in the tables.*

Thank you for this comment, we now added this information throughout the manuscripts.

*2) The name of the outcome measures (tests) should be specified before using abbreviations.*
Thank you for this comment, we now added this information.

Reviewer #2: Thank you very much for the opportunity to review the proposed manuscript.  The investigators aimed to support affected people in developing coping strategies against major PCS symptoms such as fatigue and cognitive impairments and thus improve their quality of life and social participation.
I have a few concerns with the proposed work.

*1.       In the Introduction should be added that* *there are different definitions of long COVID, referred to as Post-acute COVID-19 syndrome (PACS) or Post-acute Sequelae of SARS-CoV-w (PASC). They vary but are characterized by persistent symptoms and/or delayed or long-term complications persisting or beginning beyond four weeks from the symptom onset of a SARS-CoV-2 infection.*

Thank you, we added this point to the introduction.

*2.      As the journal is dedicated to medical professions it is worth mentioning how many respondents had been vaccinated with at least one dose of the anti-SARS-CoV-2 vaccine, and how many of participants had become unwell before the vaccine.*

It is correct that COVID-19 vaccination impacts the incidence of PCS (e.g. 10.1016/S2213-2600(24)00082-1). However, since we only included people with PCS into our study, we did not analyze vaccination status upon study inclusion.

*3.      What was the average time from the onset of the disease in people qualified according to the inclusion criteria*

Thank you for this comment. The mean time between the infection and study start is reported in Table 1 with the patient characteristics.

*4.      Divide the outcomes into primary and secondary outcomes.*

Thank you, we added clear information on primary and secondary outcomes in the new version of our manuscript.

*5.      Please discuss the topic of different programs used in Post-COVID Survivors.
More in the papers:*
[*https://doi.org/10.3390/jcm13061712*](https://doi.org/10.3390/jcm13061712) *Resistance Exercise Program Is Feasible and Effective in Improving Functional Strength in Post-COVID Survivors*
[*https://doi.org/10.3390/jcm13102747*](https://doi.org/10.3390/jcm13102747) *The Impact of Resistance Training on Equilibrium Abilities and Quality of Life in Older Adults after SARS-CoV-2 Survival*
We added information on these studies to the discussion.

Reviewer #3: Summary of the Research & Overall Impression:
This manuscript is one of the first randomized controlled trials to investigate and support positive treatment effects of individualized 1:1 Occupational Therapy intervention for individuals with post covid syndrome facing severe fatigue and cognitive impairment impacting daily functioning and participation. Additionally, this research supports the effectiveness of delivering individual occupational therapy services via telehealth virtual platforms- which has becoming increasingly popular through the COVID-19 pandemic and has implications for support current practice and accessibility of services to rural regions and for those who remain home bound due to symptom burden. Both of these topics are highly relevant to support emerging clinical practices within occupational therapy and rehabilitation sciences. Both of these topics are highly relevant to support emerging clinical occupational therapy practice to improve recovery trajectories for individuals facing post covid
conditions.  In the absences of proven drug therapies to treat the post-viral sequelae impacting a large portion of the global population across the lifespan, it is critical for research to focus on rehabilitation and supportive interventions to address the symptom burden, functional participation abilities, and satisfaction/quality of life for individuals with this condition.

Research Question: For individuals with PCS experiencing severe fatigue and cognitive dysfunction, is virtual interactive Occupational Therapy intervention more effective in improving symptoms, participation, cognitive function, and quality of life compared to pre-recorded educational videos or no intervention?

The results provide a substantial advance over existing knowledge, with clear implications for patient care, public policy, and clinical research agendas; additionally, this paper will be of interest to patients, clinicians and policymakers who are not specialists in this topic.

Major Issues to be Addressed by the Authors:
*1. The background would benefit from further connection to existing relevant literature related to post-covid symptom burden and the impact on functional participation for those affected. Consider incorporating examples on restricted ability to maintain full time employment, or participation restrictions in domains of fitness, education, or social/community routines. Consider defining Occupational Performance or "Occupations" for multidisciplinary audience- and making an explicit connection as to how* *post covid symptoms of fatigue and cognitive impairment negatively impact occupational performance abilities and satisfaction across multiple domains.*
Thank you for this helpful comment which allows us to futher illustrate the relevance of our work. We added a respective sentence to the discussion.

*2. When describing the justification for use of digital remote treatment, consider citing reasons that relate to use in* *chronic fatigue syndrome populations, remote/rural populations; or restricted transportation related to symptom burden for in-person care. Explicitly reference ability to participate in biweekly sessions for 12 weeks- and accessibility of care. Although mobility limitations is a reasonable justification, it is not connected to any of end points mentioned in the study. Some may advocate that when mobility restriction is present, in-person services may be warranted (or a hybrid model) to ensure holistic assessment and treatment needs are addressed.*
We thank the reviewer for these comments and modified our introduction and discussion accordingly.

*3. Current presentation of primary and secondary end points of the study and methods of assessing end points is challenging to follow with variable terminology used across the Methods and Results sections. For example, terminology presented in Figure 1 is not present in the Methods or Results section of the paper (Patient reported outcome measure). Consider re-structuring presentation of assessment tools and outcome measures used within the methods sections to improve clarity of reporting findings in the Results. From my multiple readings of the paper, I was able to group into 4 distinct categories (primarily based on results section): #1. Feasibility and Acceptability of Treatment Concept (Likert Scale questionnaire, drop out rates, personal OT experiences); #2. Patient Reported Outcome Measures of cognitive performance in everyday life, HRQOL, and social participation (Neuro-QOL, HRQOL, EQ-5D-3L index and EQ VAS, IMET). #3. Psychological Assessments of Memory function via
WIT-2 and Concentration via D2-R; and #4. Occupational Performance Assessment using COPM. Readers will benefit from reorganization of reporting these tools in the Methods section. A more thorough description of the COPM and the multiple measurements it obtains (patient satisfaction rating with performance, patient performance rating) is needed. Differentiate the various measures used to assess cognitive performance and consider using a consistent descriptor that differentiates the two types of assessments (self-report Neuro QOL versus Psychological assessments of memory and concentration)- make it clear to the reader why results were variable for different cognitive assessment measures across different arms of the study. The paper left me wondering- how does the Neuro-QOL tool differ from the WIT-2 and D2-R and why did they capture cognitive performance improvements differently across groups? I imagine this may relates to the cognitive rehabilitation tools (i.e.
metacognitive strategy training) provided in OT individualized care- but this is not obvious to lay person.*
We thank the reviewer for these comments. As a similar point was made by another reviewer, we revised the manuscript accordingly and added subheadings and sorted and explained the description of results in a more harmonized fashion.

*4. Reference List citations require attention for formatting.*
We modified the reference list, hopefully correcting any errors.

*5. Will supplemental materials regarding specifics of OT individual interventions be provided for readers?*
A similar point was made by another reviewer. We added more information to the methods section and cited additional papers providing more background on the OT intervention.

*6. The manuscript will benefit from copy editing for grammar, spelling, and typos.*
We apologize for any errors and hopefully corrected them with help of a native speaker.

Reviewer #4: Alex McConnachie, Statistical Review

Schröder et al present the results of a randomised trial of occupational therapy delivered online, either in person or using videos, for people with Long COVID. This review considers the statistical aspects of the paper. Overall, I think this is a decent study, and the data do suggest that the interactive version of the intervention may be beneficial, though whether this is due to the occupational therapy, or simply an attention effect, could be debated. In general, the statistical methods seem reasonable, but I have a few comments that may need attention.

*One of the main issues is the differential loss to follow-up, with more than half of the video group not completing the study. This fits with the general narrative, however, with the video-based intervention seemingly less effective than the interactive version. I would assume a video intervention to be less engaging than an in-person one, with disengagement leading to withdrawal for a proportion of participants.
Use of LOCF for missing outcome data is not ideal since this assumes no change in outcomes for those who withdraw, which is a strong assumption. Some people might have withdrawn because they felt better - the fact is there is no way of knowing. I would rather see the actual data presented, with any imputation analyses done as sensitivity analyses. Rather than LOCF, a multiple imputation approach might be better.*
Thank you for this comment. The use of LOCF is controversial, which is why we now have included the results between the groups without LOCF-approach to the supplement. In terms of outcome, there were no significant differences with vs. without LOCF-approach. Since we observed an improvement over time in all study groups (including the control group), we decided to present the LOCF method in the main body of our manuscript because it is a conservative approach. We added a respective part to the discussion.

*The main focus of the analysis appears to be in the within-group changes over time, with the between-group comparisons put into the supplement. Again, I feel this is the wrong way round - for an RCT, I would rather see the between-group differences first, with the within-group changes as supporting information. Whilst few of the between-group comparisons are statistically significant, this is not surprising for an underpowered pilot trial. Saying that, the authors do not help themselves by applying a p-value correction for multiple testing. I would say this is not necessary for a pilot trial where strong control of type I error is not the objective. The aim is to look for patterns of potential intervention effects across a range of outcomes, so a more relaxed approach is acceptable in my view.*
Thank you for this comment. We have adjusted the order of the results and added a table with the differences between groups to the main text. We would like to keep the adjustment of the p-values, as this could be noted by other reviewers. The absolute change in the effects for e.g. a sample size calculation does not change relevantly.

*The between-group comparisons are reported in Supplementary Table 1, but this is not signposted in the paper - the only reference to this table is in relation to baseline sociodemographic characteristics, which are not what this table is showing. This needs to be fixed.*
We appreciate your comment. We have restructured the results section and added the differences between groups as a table to the main text.

*I note that the analysis as described in the protocol does not talk about within-group changes, LOCF, or p-value adjustments. Was this change in emphasis pre-specified in an analysis plan?*
Thank you, that's right. We decided to perform exploratory analyses of the differences between groups, because large differences within groups were observed which was not excepted for this pilot trial. All analysis were approved by regulatory authorities.

*I have some concerns about the timing of the assessments. Figure 1 places the baseline assessments after randomisation, when they should be done before, or at least before the participants are aware of their allocation. Saying that, it is not completely clear when the baseline assessments were done. In the text, it is explicitly stated that the COPM was done after randomisation, but it is not obvious for the other measures. The protocol suggests that baseline assessments came before randomisation.*
Cognitive tests were performed before randomization. For organizational reasons, COPM was performed after randomization. We now added this information to the methodology section and also discussed this fact in the “limitations” part of our manuscript.

*The protocol suggests that the aim was to recruit from two distinct age groups (16-18, and 30-50) but this is not clear in the paper. Similarly, the protocol suggests that the randomisation was stratified by age group, but this is not evident from the paper. Also, if the study did recruit in these two separate age bands, then it would suggest that the data ought to be looked at in this way. Were the same trends seen in both age groups?*
Initially, we planned to include many adolescents and young adults into our study. However, people with PCS in this age group were not interested to participate in our study - in spite of many efforts to recruit them. We changed our study protocol accordingly focused on the patient group with most immanent clinical need (adults and seniors). All analysis were approved by regulatory authorities.

*It is stated that participants declared written consent. What does this mean, if everything was done online?*
Consent was given online through a digital signature. This information is now added to the text.

*There was no sample size calculation, which is fine for a pilot trial, but what was the target? The protocol states 160, but this is not quoted in the paper, so it is not possible to say whether the trial achieved its target (apparently it did).*
Thanks for the comment. It is true that the planned recruitment number of 160 subjects is not mentioned in the text. We have updated this information.

*It is generally not advised to report p-values for comparisons of baseline characteristics in RCTs (Table 1).*
Thank you for your this comment. We corrected this error.

*Table 1 reports 136 female participants, but the numbers in the randomised groups add up to 92, and the percentages do not look right. Something has gone wrong here.*
Thank you for noticing this mistake. We have adjusted the values for gender.

*The title of Table 2 is "Effects of the study intervention on…" which is not right - these are within-group changes, and cannot be interpreted as intervention effects.*
Thank you for this comment. We changed the title of this table to: “Within group changes on cognitive performance, quality of life, and occupational performance”
