# Supplementary material for: Improvement in quality of life and cognitive function in Post-COVID syndrome after online occupational therapy: Results from a randomized controlled pilot study
Source: PLoS One. 2025 May 20;20(5):e0312714. doi: 10.1371/journal.pone.0312714 (PMC12091760; doi:10.1371/journal.pone.0312714)
Supplement: Attachment — Submitted filename: Reviewer_pointbypoint.docx [file pone.0312714.s007.docx]

Reviewer #1:

We would like to thank reviewer #1 for their work in assessing our manuscript. We have addressed the remaining concerns as stated below:

Table 2 title: The abbreviation 'LOCF' is to be replaced.

We have replaced the abbreviation ‘LOCF’ with ‘multiple imputations’ to remove the need for an abbreviation in the title.
Line 165: To state the assumptions for repeated measure ANOVA were fulfilled prior to use.

We now clarify that we tested our data for conforming to the underlying assumptions on lines 165 and 166.

Reviewer #2: A nice job ready for publication after address the two minor points raised by Reviewer 1, thank you all authors.

We thank reviewer #2 for their help in assessing and improving our manuscript!
